# Fatty liver index is a strong predictor of changes in glycemic status in people with prediabetes: The IT-DIAB study

**DOI:** 10.1371/journal.pone.0221524

**Published:** 2019-08-29

**Authors:** Matthieu Wargny, Sarra Smati, Matthieu Pichelin, Edith Bigot-Corbel, Charlotte Authier, Violette Dierry, Yassine Zaïr, Vincent Jacquin, Samy Hadjadj, Jérôme Boursier, Bertrand Cariou

**Affiliations:** 1 L’institut du thorax, Department of Endocrinology, CIC 1413 INSERM, CHU Nantes, Nantes, France; 2 L’institut du thorax, INSERM, CNRS, UNIV NANTES, CHU Nantes, Nantes, France; 3 La Clinique des Données, CHU Nantes, Nantes, France; 4 Department of Biochemistry, CHU de Nantes, Nantes, France; 5 Centre d’examens de la Caisse Primaire d’Assurance Maladie de Loire-Atlantique, Saint-Nazaire, France; 6 Hepatology Department, Angers University Hospital, Angers, France; HIFIH Laboratory, UPRES 3859, SFR 4208, Angers University, Angers, France; INRA, FRANCE

## Abstract

**Background & aims:**

In patients at metabolic risk, nonalcoholic fatty liver disease is a strong and highly prevalent predictor for type 2 diabetes. Its assessment in clinical practice is not easy but the fatty liver index (FLI) could be used as a surrogate. Here, we studied the association between the FLI and the conversion to new-onset diabetes (NOD) or prediabetes reversion in patients with prediabetes.

**Methods:**

The IT-DIAB observational study included 389 individuals with prediabetes, defined as fasting plasma glucose (FPG) between 110 and 125 mg/dL. NOD conversion was defined as a first FPG value ≥ 126 mg/dL and prediabetes reversion as a first FPG value < 110 mg/dL. The associations of both events with baseline FLI were studied separately using multivariate Cox models.

**Results:**

After a median follow-up of 3.9 years (range 0.1–6.1), 138 individuals (35.5%) converted to NOD. FLI was associated with a higher risk of NOD conversion (unadjusted HR per SD = 1.54, _95%_CI 1.27–1.86, p<0.0001), even after multiple adjustment on FPG, HbA_1c_ and diabetes risk score (adjusted HR per SD 1.31, _95%_CI 1.07–1.61, p = 0.008). FLI was also associated with prediabetes reversion: adjusted HR per SD = 0.85, _95%_CI 0.75–0.96, p = 0.0077. Changes in FLI were significantly associated with changes in FPG during follow-up (p<0.0001). When compared to a full model including the diabetes risk score, FPG, HbA_1C_ and FLI, only HbA_1C_ added a significant prediction information (AUROC: 72.8% for full model vs 69.4% for the model without HbA_1C_; p = 0.028), while the removal of FLI to the full model did not alter its predictive value (AUROC 72.2%). The predictive value for NOD conversion was not significantly better for HOMA-IR compared to FLI (AUROC: 69.3 vs 63.7%, p = 0.067).

**Conclusions:**

FLI is a simple, practical score to further stratify the risk of conversion to NOD or the possibility of prediabetes reversion in clinical practice, independently of classical glucose parameters.

**Trial registration:**

ClincialTrials.gov number NCT01218061 and NCT01432509.

## Introduction

Nonalcoholic fatty liver disease (NAFLD) is the most common liver disease worldwide. It has been estimated that about 25% of adults in the United States and Europe are affected by NAFLD [1]. The epidemiology of NAFLD parallels the pandemic of obesity since NAFLD is strongly associated with visceral obesity and the metabolic syndrome (MetS) [2]. In a recent meta-analysis, more than 50% (51.3%) of individuals with NAFLD are overweight or obese and around a quarter (22.5%) have a diagnosis of type 2 diabetes (T2D) [3]. Thus, NAFLD can be considered as the hepatic feature of the MetS.

NAFLD and the MetS share some common pathophysiological pathways [2]. Hepatic insulin resistance leads to an increase in hepatic glucose production, *de novo* lipogenesis and hepatic production of triglyceride (TG)-rich VLDL particles. Peripheral insulin resistance in adipose tissue promotes lipolysis and the release of free fatty acids (FFA) in the bloodstream that are taken up by the liver to promote hepatic steatosis [4]. Liver fat is increased in people with MetS compared with those without, and all components of the MetS are significantly correlated with liver fat content [5]. Importantly, concordant observational studies suggest that NAFLD is a predictor of metabolic diseases, such as T2D [6]. For instance, a study has demonstrated that liver steatosis assessed by abdominal ultrasonography predicts NOD independently of all markers of the MetS [7]. Beyond T2D, consistent studies have shown that NAFLD is also an independent risk factor for cardiovascular diseases [8].

There is a growing need for a wide screening for NAFLD in patients at metabolic risk in order to promote an individualized life style management and an early detection of metabolic complications. Due to the high prevalence of NAFLD in the general population, a simple surrogate marker to screen for NAFLD in clinical practice is warranted. The FLI is a simple index constructed using measures of TG, gamma glutamyl-transferase (GGT), body mass index (BMI) and waist circumference [9]. FLI has been shown to be an accurate surrogate marker of hepatic steatosis in Asian and Western populations [10,11]. FLI correlates with insulin resistance [12] and was recently shown to predict NOD in subjects with prediabetes in Asian [13,14] and in European populations [15].

Here, we aimed to determine the clinical interest of using FLI to predict NOD conversion, but also prediabetes reversion, in individuals with prediabetes in the 5-year longitudinal IT-DIAB study. We also investigated whether the changes in FLI are associated to changes in FPG in this non-interventional study.

## Materials and methods

### Study population

The IT-DIAB study, including Therapeutic Innovation in Type 2 DIABetes (NCT01218061) and DiabeNord (NCT01432509) trials, was designed to identify new biomarkers of T2D risk in a population with prediabetes. It is a 5-year, prospective, observational study carried out in occupational centres based in three French cities: Nantes, Saint-Nazaire and Lille. From June 2010 to February 2013, we included 430 patients aged 18 and over presenting with impaired fasting glucose (IFG) defined as FPG ≥110 and <126 mg/dL.

Main non-inclusion criteria were a history of treatment with oral anti-diabetic agent or insulin (except for gestational diabetes), severe coagulation disorders or thrombocytopenia, severe renal insufficiency (defined using MDRD equation as eGFR<30 mL/min.1.73m^2^), severe liver impairment (prothrombin ratio <50%), severe psychiatric disorders, alcohol abuse estimated >30 g/day, patient’s opposition or inability to follow the study at least 5 years.

### Data collection

At baseline, all patients underwent medical examination, and fasting blood sample was taken for biological analysis and the constitution of a biocollection. The medical examination included a standard questionnaire (lifestyle, alcohol and smoking habits, occupation, diabetes risk score [16], current medication) and anthropometric measurements (weight, height, waist and hip circumferences, systolic/diastolic blood pressure). Biological analyses were performed in certified medical analyses laboratories and included FPG, HbA_1c_, lipid profile (total cholesterol, TG and HDL-C, the LDL-C being calculated using Friedewald’s formula), liver profile (AST, ALT, GGT) and creatinine. This process was repeated every year.

Also, insulin was determined centrally (CHU Nantes) from frozen heparinized plasma collected in the centres of IT-DIAB trial (Nantes and Saint-Nazaire) using Cobas e automated clinical analyzer system (Roche Diagnostics, Meylan, France) by electrochemiluminescent enzyme immunoassay (ECLIA). Likewise, plasma high molecular weight adiponectin levels were centrally (CHU Nantes) measured by ECLIA on an automated clinical analyzer system Lumipulse G600 (Fujirebio, Les Ulis, France).

Patients’ follow-up was originally planned for 5 years or until the conversion to NOD, introduction of an oral anti-diabetic agent or insulin therapy before diabetes occurrence, bariatric surgery, patient withdrawal or lost from sight or death.

### Primary outcomes definition and other parameters of interest

The primary outcome was the conversion to NOD, defined by a first FPG value ≥126 mg/dL and/or 2H-glycemia ≥200 mg/dL during OGTT (Oral Glucose Tolerance Test), whichever came first. For the purpose of sensitivity analyses, we defined “confirmed NOD conversion” as a NOD conversion confirmed by a second FPG value ≥126 mg/dL before 1 year. Prediabetes reversion was defined as a first FPG value <110 mg/dL. The FLI was calculated using the formula proposed by Bedogni based on BMI, waist circumference, TG and GGT [9]. HOMA-IR (Homeostasis model assessment of insulin resistance) was defined according to the equation proposed by Matthews et al [17]. MetS was defined according to IDF (International Diabetes Federation) consensus statement [18].

### Informed consent/ethic committee and authors approval

The IT-DIAB study protocol was approved by the Ethic Committee of Tours University Hospital (CPP “Ouest I”, approval n°2010-S2); the DiabeNord study protocol was approved by the Ethic Committee of Amiens University Hospital (CPP “Nord-Ouest II”, approval n°2011/24). All patients received oral information by the investigators and signed an informed consent before any study procedure.

All the co-authors had access to the study data and reviewed and approved the final manuscript.

### Statistical analyses

Data analyses were restricted to the population with available FLI data at baseline and at least one follow-up visit. We described the study population according to the diabetes status at the end of follow-up (conversion to NOD or not) using classical description parameters (population size (%), mean ± standard deviations or median (25^th^-75^th^ percentiles), according to the distribution). For proportions, 95% confidence intervals (95% CI) were assessed using the Wilson score method. The Kaplan-Meier survival curves for conversion to NOD were presented according to FLI value using 30 and 60 as thresholds [10]. Their association was then studied using multivariate Cox model adjusted on age, sex, diabetes risk score, the presence of hypertension, the use of statin therapy, and baseline FPG and HbA_1c_, before and after a stepwise selection process using a p-value threshold = 0.05. Multivariate fractional polynomial degree 2 models were tested (model selection threshold = 0.20) to account for the possibility of non-linear relationships. The study of the association between FLI and prediabetes reversion was conducted likewise.

We performed different sensitivity analyses: (i) To compare FLI and HOMA-IR, the same Cox models were built in the subsample with available HOMA-IR values at baseline; (ii) To define conversion to NOD according to the American Diabetes Association (ADA) classification [19], we performed Cox analyses after the exclusion of the population with baseline HbA_1c_ ≥6.5%, and added this criterion in the definition of conversion to NOD; (iii) lastly, we repeated the same analysis process considering confirmed NOD conversion as the primary outcome.

All analyses were performed using R software (version 3.5, “R core group”, Vienna) with the packages *ggplot2* and *mfp*. A p-value <0.05 was deemed statistically significant.

## Results

### IT-DIAB population and follow-up

**Fig 1:**
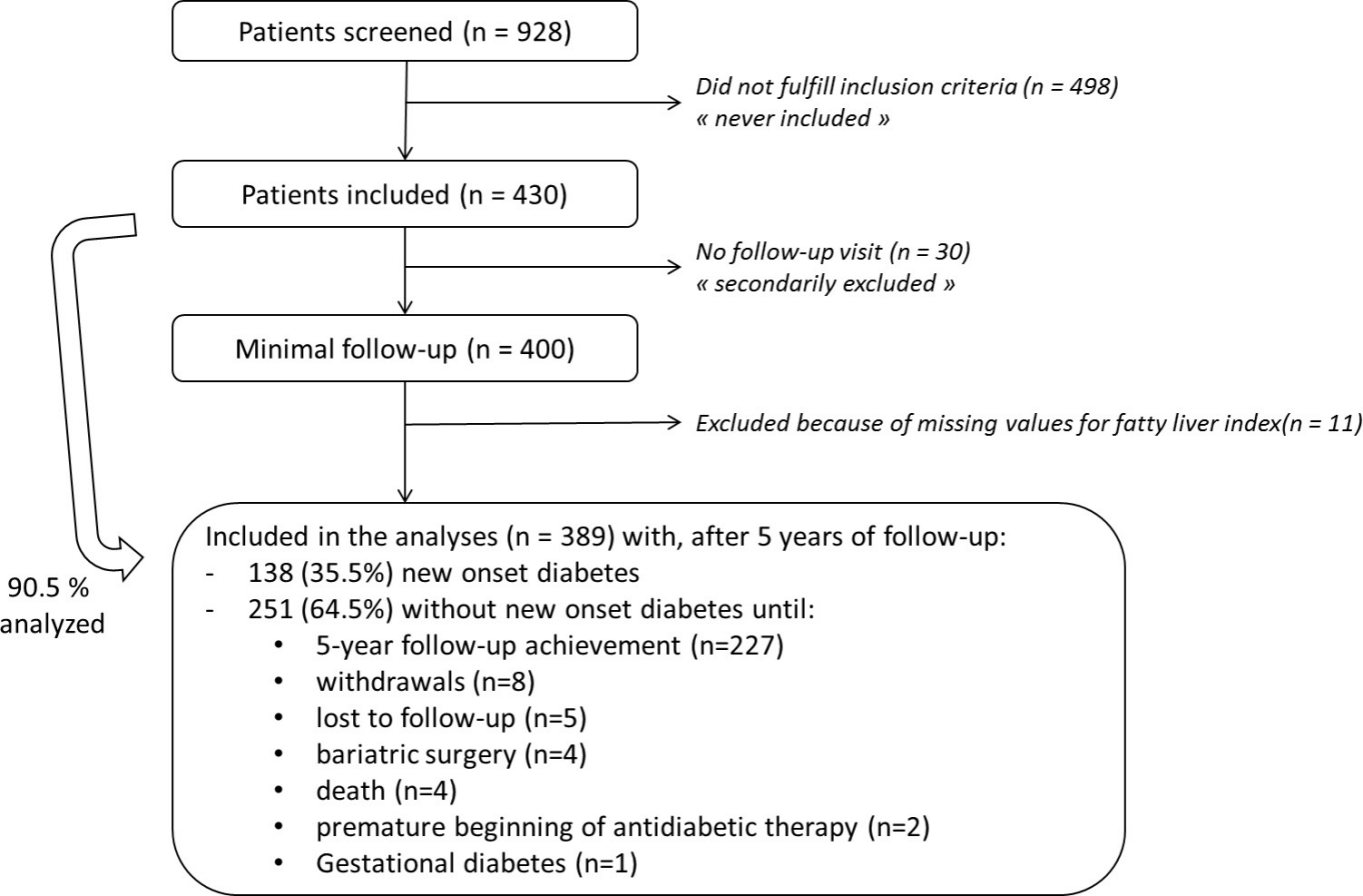
IT-DIAB study flowchart.

The flow chart of the study was shown in Fig 1. In the original 430 individuals with IFG included in the IT-DIAB study, 30 patients (7%) never showed up for follow-up visits and 11 patients (2.6%) had missing baseline FLI value. Therefore, 389 (90.5%) were finally included in the statistical analyses. Characteristics of IT DIAB participants are detailed in the S1 Table. The median follow-up was 3.9 years (0.1–6.1 years), during which 138 people (35.5%) converted to NOD.

### Conversion to NOD compared to those remaining free of diabetes

Baseline characteristics of the patients according to diabetes status at the end of follow-up are listed in Table 1. Briefly, compared to those remaining free of diabetes, patients with conversion to NOD during the follow-up had higher BMI, waist circumference and frequency of MetS (66.4% vs 43.6%). As expected, they also had a higher HOMA-IR and lower adiponectin levels indicating a more insulin-resistant phenotype. Regarding the lipid profile, subjects with conversion to NOD had increased TG and reduced HDL-C levels. While liver enzymes (AST, ALT and GGT) were slightly higher, the FLI was more clearly increased (73.7 *vs* 59.4) in the conversion to NOD group. Similar results, with sometimes more pronounced differences, were found in the subgroup of patients with confirmed NOD conversion.

**Table 1:** Baseline characteristics of the IT-DIAB population (n = 389) according to the final status for conversion to new onset diabetes.

	No new onset diabetes n = 251	Conversion to new onset diabetes n = 138	Confirmed conversion to new onset diabetes (n = 41)
*Clinical*			
Women	71 (28.3%)	46 (33.3%)	13 (31.7%)
Age (years)	58.4 ± 10.3	57.6 ± 8.8	57.9 ± 9.3
Diabetes Risk Score	13.3 ± 4.6	15.7 ± 4.0	16.9 ± 3.6
Body mass index (kg/m^2^)	28.4 ± 5.2	31.2 ± 6.0	33.0 ± 7.2
Waist circumference (cm)	96.8 ± 13.2	102.8 ± 14.3	106.4 ± 14.9
Hip circumference (cm)	101.8 ± 10.0	107.3 ± 14.7	110.5 ± 17.9
Waist/Hip ratio	0.95 ± 0.09	0.96 ± 0.09	0.97 ± 0.09
Hypertension	96 (38.4%)	62 (44.9%)	19 (46.3%)
Metabolic syndrome^**†**^	109 (43.6%)	91 (66.4%)	30 (73.2%)
Statin therapy	60 (23.9%)	35 (25.4%)	12 (29.3%)
*Biological (fasting)*			
Glucose homeostasis			
Plasma glucose (mg/dL)	115 (112–119)	117 (113–121)	120 (114–123)
HbA1c (%)	5.8 ± 0.4	6.0 ± 0.4	5.9 ± 0.4
HbA_1c_ (mmol/mol)	39.4 ± 4.2	41.7 ± 4.6	40.6 ± 4.0
Fasting serum insulin (mUI/L)	12.2 ± 8.1	17.3 ± 10	17.0 ± 10.0
HOMA-IR	3.42 ± 2.41	5.08 ± 2.92	4.94 ± 2.83
HMW-Adiponectin (μg/mL)	3.94 ± 2.30	3.29 ± 1.93	3.40 ± 1.53
Liver enzymes			
AST (UI/L)	21.7 ± 7.9	25.2 ± 11.4	28.1 ± 15.2
ALT (UI/L)	30.1 ± 17.2	34.1 ± 20.1	38.4 ± 27.4
GGT (UI/L)	45.0 ± 42.7	46.1 ± 35.0	52.6 ± 38
Lipid profile			
Total cholesterol (mg/dL)	215.5 ± 37.8	213.3 ± 40.3	206 ± 41.3
Triglycerides (mg/dL)	126.2 ± 76.8	144.5 ± 75.6	144.8 ± 74.1
LDL-C (mg/dL)	135.6 ± 35	134.3 ± 35.1	126.8 ± 37.3
HDL-C (mg/dL)	54.6 ± 15.6	50.3 ± 12.6	50.0 ± 15.0
Non-HDL-C (mg/dL)	160.9 ± 40.1	162.5 ± 38.7	156 ± 41.6
*Fatty liver index*			
Raw value	59.4 (30.7–79.9)	73.7 (47.4–93.2)	83.6 (59.9–95.2)
<30	62 (24.7%)	15 (10.9%)	2 (4.9%)
30–59	65 (25.9%)	38 (27.5%)	9 (22%)
≥ 60	124 (49.4%)	85 (61.6%)	30 (73.2%)

^**†**^ According to the International Diabetes Federation consensus statement.

HOMA-IR: Homeostasis model assessment of insulin resistance; HMW: High Molecular Weight

Baseline clinical and biological characteristics according to FLI are shown in the S1 Table.

### Predicting factors of conversion to NOD

**Fig 2:**
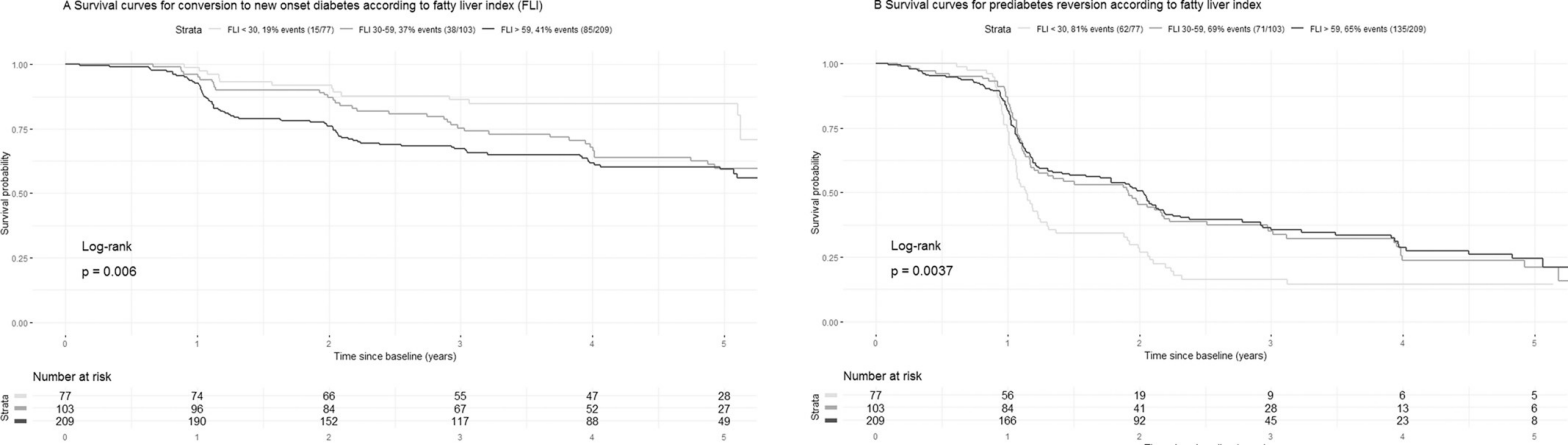
Survival curves according to fatty liver index: conversion to new onset diabetes (A) and prediabetes reversion (B).

A FLI value <30 was associated with a 19.5% probability (95% CI, 11.3–30.1) of conversion to NOD, compared to 36.9% (95% CI, 27.6–47.0) for a FLI value 30–59, and 40.7% (95% CI, 33.9–47.7) with FLI ≥60. The survival curves showing the 3 subsets of population built using these FLI thresholds pointed for earlier conversion to NOD associated with highest FLI values: 5 conversions to NOD (6.5%) after 1 year of follow-up for the group with FLI < 30, 10 (9.7%) for the group with FLI 30–59 and 43 (21%) for the group with FLI ≥ 60 (Fig 2A).

Clinical and biological variables associated with conversion to NOD using univariate Cox models are detailed in Table 2. Briefly, diabetes risk score, BMI, waist circumference, MetS, AST concentration, FLI and HOMA-IR were positively associated with conversion to NOD.

**Table 2:** Baseline characteristics of the population associated with conversion to new onset diabetes and with prediabetes reversion—Univariate Cox model.

	Cox model–Univariate analyses					
	Conversion to new onset diabetes (138 events/389)			Prediabetes reversion (268 events/389)		
	HR [95% CI]	*p*-value	Event/ pop. size	HR [95% CI]	*p*-value	Event/ pop. size
Clinical characteristics						
*Age (+1 SD)*	0.96 [0.81; 1.13]	0.60	138/389	0.93 [0.82; 1.05]	0.24	268/389
*Sex (Women/men)*	1.19 [0.83; 1.70]	0.34	138/389	1.08 [0.83; 1.41]	0.57	268/389
*Diabetes risk score (+1 SD)*	1.50 [1.26; 1.78]***	**<0.0001**	138/389	0.84 [0.74; 0.95]**	**0.0047**	268/389
*Body mass index (+1 SD)*	1.39 [1.22; 1.58]***	**<0.0001**	138/389	0.81 [0.71; 0.94]**	**0.0036**	268/389
*Waist circumference (+1 SD)*		**<0.0001**	138/389		0.29	268/389
*For men*	1.92 [1.54; 2.40]***			0.74 [0.62 ; 0.88]		
*For women*	**1.19 [0.97 ; 1.47]**			0.85 [0.69 ; 1.05]		
*Hip circumference (+1 SD)*	1.37 [1.19; 1.57]***	**<0.0001**	136/385	0.89 [0.78; 1.01]	0.069	266/385
*Waist/hip ratio (+1 SD)*	1.21 [1.03; 1.43]*	**0.019**	136/385	0.82 [0.72; 0.92]**	**0.0014**	266/385
*Hypertension (y/n)*	1.31 [0.94; 1.83]	0.12	138/388	0.91 [0.71; 1.17]	0.47	267/388
*Metabolic syndrome*^**†**^ *(y/n)*	2.30 [1.61; 3.28]***	**<0.0001**	137/387	0.73 [0.57; 0.93]**	**0.0098**	266/387
*Statin therapy (y/n)*	1.24 [0.84; 1.83]	0.27	138/389	0.80 [0.59; 1.07]	0.13	268/389
Liver associated biomarkers						
*AST (+1 SD)*	1.31 [1.10; 1.56]**	**0.0027**	89/244	0.98 [0.84; 1.15]	0.81	152/244
*ALT (+1 SD)*	1.14 [0.99; 1.31]	0.062	138/388	0.95 [0.84; 1.08]	0.42	267/388
*GGT (+1 SD)*	1.04 [0.89; 1.22]	0.61	138/389	1.13 [0.99; 1.28]	0.068	268/389
*Fatty Liver Index (+1 SD)*	1.54 [1.27; 1.86]***	**<0.0001**	138/389	0.84 [0.74; 0.94]**	**0.0037**	268/389
Other biomarkers						
*Fasting plasma glucose (+1 SD)*	1.49 [1.26; 1.77]***	**<0.0001**	138/389	0.72 [0.63; 0.82]***	**<0.0001**	268/389
*HbA*_*1c*_ *(+1 SD)*	1.62 [1.35; 1.95]***	**<0.0001**	135/384	0.76 [0.67; 0.87]***	**<0.0001**	266/384
*Fasting insulin (+1 SD)*	1.38 [1.18; 1.61]***	**<0.0001**	100/275	0.89 [0.77; 1.03]	0.12	210/275
*HOMA-IR (+1 SD)*	1.42 [1.23; 1.66]***	**<0.0001**	100/275	0.86 [0.74; 0.99]*	**0.042**	210/275
*HMW-Adiponectin (+1 SD)*	0.80 [0.64; 1.01]	0.056	100/276	1.05 [0.92; 1.21]	0.47	210/276
*Total cholesterol (+1 SD)*	0.90 [0.76; 1.07]	0.23	138/389	1.06 [0.94; 1.19]	0.36	268/389
*TG (+1 SD)*	1.16 [1.01; 1.33]*	**0.037**	138/389	0.99 [0.87; 1.12]	0.86	268/389
*LDL-C (+1 SD)*	0.91 [0.77; 1.08]	0.29	136/384	1.02 [0.90; 1.15]	0.74	263/384
*HDL-C (+1 SD)*	0.77 [0.65; 0.93]**	**0.0063**	137/388	1.13 [1.00; 1.27]	0.054	267/388
*Non-HDL-C (+1 SD)*	0.98 [0.83; 1.16]	0.81	137/388	1.01 [0.90; 1.14]	0.86	267/388

^**†**^ According to the IDF consensus statement

HOMA-IR: Homeostasis model assessment of insulin resistance; HMW: High Molecular Weight; HR: Hazard-ratio

* *p* <0.05;

** *p* < 0.01;

*** *p* < 0.001

In multivariate Cox models, after stepwise selection, the factors associated with the risk of conversion to NOD were the Diabetes risk score (HR per SD = 1.31, 1.07–1.60), FPG (HR per SD = 1.46, 1.23–1.74), HbA_1C_ (HR per SD = 1.52, 1.25–1.84), and FLI (HR per SD = 1.31, 1.07–1.61) (Table 3).

**Table 3:** Baseline characteristics of the population associated with conversion to new onset diabetes and with prediabetes reversion, survival approach—Multivariate Cox analysis.

	Multivariate Cox analysis			
	**Before selection**		**After stepwise selection**^**†**^	
	**HR [95% CI]**	***p*-value**	**HR [95% CI]**	***p*-value**
Study of conversion to new onset diabetes (135 events/383)				
*Age (+1 SD)*	0.93 [0.77; 1.12]	0.43	-	-
*Sex (Women/men)*	0.90 [0.60; 1.34]	0.59	-	-
*Diabetes risk score (+1 SD)*	1.35 [1.08; 1.68]**	**0.0081**	1.31 [1.07; 1.60]**	**0.0089**
*Hypertension (y/n)*	1.00 [0.67; 1.47]	0.98	-	-
*Statin therapy (y/n)*	0.92 [0.61; 1.40]	0.71	-	-
*Fasting plasma glucose (+1 SD)*	1.47 [1.23; 1.75]***	**<0.0001**	1.46 [1.23; 1.74]***	**<0.0001**
*HbA*_*1c*_ *(+1 SD)*	1.54 [1.26; 1.88]***	**<0.0001**	1.52 [1.25; 1.84]***	**<0.0001**
*Fatty Liver Index (+1 SD)*	1.28 [1.03; 1.58]*	**0.024**	1.31 [1.07; 1.61]**	**0.008**
	**Before selection**		**After stepwise selection**^**†**^	
Study of prediabetes reversion (265 events/383)	**HR [95% CI]**	***p*-value**	**HR [95% CI]**	***p*-value**
*Age (+1 SD)*	0.93 [0.81; 1.06]	0.29	-	-
*Sex (Women/men)*	1.22 [0.91; 1.65]	0.19	-	-
*Diabetes risk score (+1 SD)*	0.88 [0.76; 1.03]	0.11	**-**	**-**
*Hypertension (y/n)*	1.13 [0.85; 1.51]	0.40	-	-
*Statin therapy (y/n)*	1.02 [0.74; 1.41]	0.89	-	-
*Fasting plasma glucose (+1 SD)*	0.75 [0.66; 0.86]***	**<0.0001**	0.75 [0.66; 0.86]***	**<0.0001**
*HbA*_*1c*_ *(+1 SD)*	0.79 [0.68; 0.90]***	**0.00063**	0.79 [0.69; 0.90]***	**0.0003**
*Fatty Liver Index (+1 SD)*	0.89 [0.77; 1.03]	0.11	0.85 [0.75; 0.96]**	**0.0077**

^**†**^Considered candidates for multivariate adjusted Cox model were defined as follows: baseline fasting plasma glucose and Fatty liver index were “forced” in the model; age, sex, diabetes risk score, hypertension, statin therapy, HbA_1c_ were candidates. Multivariate Fractional Polynomial approach was also considered, but no polynomial transformation was proposed for a threshold selection value = 0.20

* p <0.05;

** p < 0.01;

*** p < 0.001

The multivariate polynomial approach did not highlight significant non-linear relationship between the above factors and the risk of conversion to NOD. Also, no significant interactions were found between these factors in the different models. Particularly, no interaction was found between FLI and gender (p = 0.35).

As a next step, we performed comparisons of ROC curves for NOD conversion using different prediction models to estimate the added predictive power of each factor (S1 Fig). We defined a full model including the diabetes risk score, FPG, HbA_1C_ and FLI and observed the result of the independent removal of all the 4 components. It appears that only HbA_1C_ added a significant prediction information (AUROC: 72.8% for full model vs 69.4% for the model without HbA_1C_; p = 0.028), while the AUROC of the models deprived of diabetes Risk Score, FPG and FLI were not significantly different from the full model (AUROC 71.2, 70.3 and 72.2%, respectively).

The sensitivity analyses using the confirmed NOD conversion as the outcome of interest also showed significant associations between FLI and confirmed NOD conversion in multivariate Cox models (adjusted HR per 1 SD of FLI = 1.62 [1.08; 2.43], *p*-value = 0.020) (S2 Table). However, the sensitivity analyses including the HbA_1c_ criterion in the definition of NOD showed only a not statistically significant trend for the association between FLI and NOD conversion (adjusted HR per SD of FLI = 1.23 [0.99; 1.51], *p*-value = 0.058).

### Comparison of FLI and HOMA-IR as predictors of conversion to NOD

In the subset with available dosages of fasting insulin (n = 275), FLI and HOMA-IR were strongly correlated at baseline (corr = 0.65; p <0.0001) (S2 Fig). The Cox model including diabetes risk score, FPG, HbA_1C_, FLI and HOMA-IR showed neither significant association with conversion to NOD for HOMA-IR (HR per SD = 1.14, 95% CI 0.93–1.40), nor for FLI (HR per SD = 1.27, 95% CI 0.96–1.68). However, after the removal of FLI from the model, HOMA-IR reached significance to predict NOD conversion (HR per SD = 1.26, 95% CI 1.07–1.49). When comparing the ROC curves of HOMA-IR and FLI for the detection of the event « NOD » during follow-up, the AUC associated with HOMA-IR was substantially, but not significantly, higher (AUC 69.3% vs. 63.7%, p = 0.067) (S3 Fig).

### FLI and prediabetes reversion

We also assessed the clinical relevance of FLI for predicting prediabetes reversion. Patients with FLI <30 had a higher probability of prediabetes reversion compared to those with FLI 30–59 and FLI ≥60 (log-rank test = 0.0037) (Fig 2B).

The univariate analyses studying prediabetes reversion based on Cox models showed results mirroring what we found for conversion to NOD: compared to those who did not reverse, patients with prediabetes reversion had lower diabetes risk score, BMI, a lower prevalence of MetS, lower FLI, lower FPG, HbA_1c_ and HOMA-IR (Table 2). In multivariate analyses, FPG, HbA_1C_ and FLI (HR per SD = 0.85, 95% CI 0.75–0.96) but not the diabetes risk score were retained in the final model (Table 3).

For sensitivity analyses, when excluding the population who met both prediabetes reversion and NOD during the follow-up, the results were similar in the subset of 332 individuals (HR for prediabetes reversion, per 1 SD of FLI = 0.83 [0.72; 0.95]).

### Covariation of FLI and FPG during follow-up

**Fig 3:**
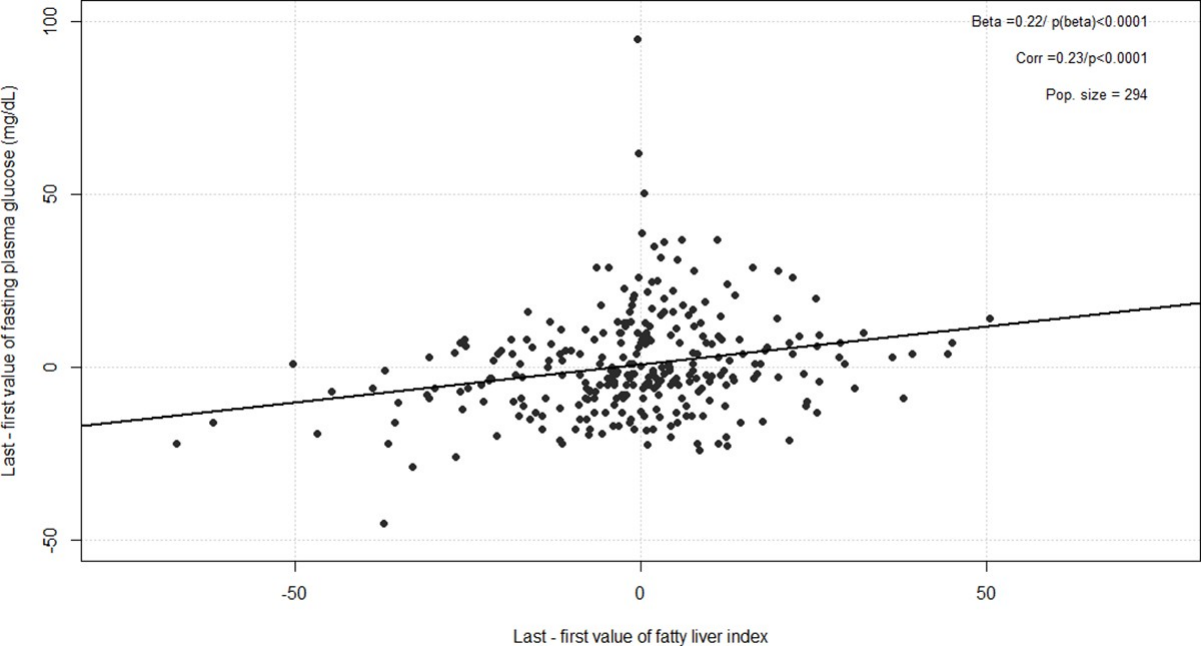
Covariation of fatty liver index and fasting plasma glucose between baseline and final visit.

At the end of the follow-up, the change of value for FPG and FLI showed significant correlation, with a Pearson’s correlation coefficient = 0.22 (p <0.0001) (Fig 3): a raw increase of 4.5% for FLI was associated with an increase of 1 mg/dL of FPG.

## Discussion

In this study, we confirmed that FLI is a useful and simple clinical biomarker to identify individuals at risk of developing T2D. Indeed, in patients with IFG, FLI strongly predicted the risk of conversion to NOD during 5 years of follow-up, independently of traditional risk factors for diabetes such as age, gender, diabetes risk score, FPG or HbA_1C_. We also showed for the first time, to the best of our knowledge, that low baseline FLI value was independently associated with the prediabetes reversion. Finally, we found that changes in FLI were correlated with changes in FPG during follow-up.

Previous concordant studies have highlighted the pathophysiological link between NAFLD and the risk of developing T2D [20]. However, the way to diagnose NAFLD and/or liver steatosis varies between studies. The largest population-based studies used liver enzymes such as aminotransferase (ALT) or GGT as surrogate markers of NAFLD [21–23]_,_ and other study assessed ultrasound-diagnosed NAFLD as determinant of NOD [24]. For instance, ALT predicts the risk of conversion to NOD after 20-year of follow-up in the Framingham Offspring Heart Study after adjustment for several risk factors, even when considering ALT values within the normal ranges [21].

FLI could be a suitable marker of NAFLD in clinical practice since it only requires simple anthropometric (BMI and waist circumference) and biochemical (TG and GGT) measures. It is noteworthy that previous studies have demonstrated that FLI is a good predictor of conversion to NOD both in the general population [25,26] and more recently in patients with prediabetes in Japanese [14] and Spanish [15] cohorts, with rather concordant results between the latter study and our current findings, as far as sampling and confounding parameters choice are taken into account. However, the population of IT-DIAB was apparently at greater risk for diabetes, as about 35% of subjects converted to NOD during follow-up compared to 6.0 to 10.3% in the Japanese cohort [14] and 9.4% in the PREDAPS study [15]. This difference could be potentially explained by the much lower values for BMI (median 23.6 kg/m^2^, vs. 28.1 in IT-DIAB) and waist circumference (median 85 cm vs. 98 in IT-DIAB) in the Japanese cohort even if ethnicity comparisons must be taken with great caution. Due to the design of the IT-DIAB study, with a longitudinal follow-up including annual anthropometric and biochemical parameters, we were able to draw survival curves (Fig 2) and FLI trajectories according to NOD progression (S4 Fig). The latter visually suggested an increase in FLI values in the 2 years preceding conversion to NOD. From a clinical perspective, patients who had both IFG and FLI ≥ 60% had an annual average risk of more than 7% to convert to NOD during the 5-year follow-up. In addition to FPG and HbA_1C_, FLI is thus useful in patients with prediabetes to further identify those most at risk of conversion to NOD in order to initiate primary prevention efforts with aggressive lifestyle management [27]. FLI also presents a special interest as it is not built on parameters related to glucose, at variance with HOMA-IR or Diabetes Risk Score which are, by design, associated with a higher probability to cross a higher threshold for glucose.

A major driver of the risk of T2DM in patients with NAFLD is insulin resistance [2,20]. However, measuring insulin resistance routinely in clinical practice remains a challenge. The HOMA-IR is the best surrogate marker of insulin resistance, but the cost for the determination of circulating insulin might be a barrier. In a subset of IT-DIAB study, we had the opportunity to address the performance of FLI, HOMA-IR and also plasmatic adiponectin (HMW form) levels for predicting conversion to NOD. FLI and HOMA-IR were strongly and positively correlated, as widely shown [12]. When putting the HOMA-IR in the multivariate analysis, the FLI was no longer significantly associated with conversion to NOD, indicating that its predictive value is mainly driven by its ability to capture insulin resistance. When comparing the ROC curves, there was a non-significant trend for a better performance of HOMA-IR compared to FLI for predicting conversion to NOD. Altogether, these data indicated that FLI could be an effective and efficient alternative to HOMA-IR in clinical practice to predict conversion to NOD in patients with prediabetes.

Importantly, we showed for the first time that FLI is a strong predictor for prediabetes reversion. In multivariate Cox analyses, FLI remained independently associated with the prediabetes reversion after accounting for traditional risk factors. The significant positive correlation between the variations of FLI and FPG further reinforces the hypothesis for a direct action of NAFLD on the risk of T2D. In accordance with such a dynamic relationship between NAFLD and T2D risk, a large Korean 5-year study demonstrated that change in fatty liver status assessed by ultrasound over time is associated with markedly variable risks of NOD [28]. While resolution of NAFLD was not inversely associated with NOD (OR: 0.95, _95%_CI 0.46–1.60) in this study, the risk of NOD was increased in subjects who developed NAFLD (OR: 2.49, _95%_CI 1.49–4.14) and even more in those in whom NAFLD worsened over 5 years (OR: 7.38, _95%_CI 3.36–16.22). According to our results, we can speculate that beyond its predictive value for conversion to NOD, FLI could also be used as a biomarker to assess the efficacy of therapeutic interventions initiated in at risk patients. However, whether treatments leading to improved FLI also lead to improved glucose homeostasis needs to be firmly established.

Our study presents some limitations. Regarding the definition of conversion to NOD, our primary analysis did not include the HbA_1c_ ≥6.5% criterion as recommended by the ADA [19], because the IT-DIAB population was not primitively selected on baseline HbA_1c_. For this reason, we performed sensitivity analyses using ADA definition of conversion to NOD, as well as an analysis using confirmed NOD conversion criterion, both showing similar results regarding the association between FLI and conversion to NOD. Even if the FLI was first proposed as a good readout of hepatic steatosis [9], we were not able to precisely assess hepatic steatosis in our population as relevant imaging data, US or MRI, were not available. Because of our limited sample size, we lack the statistical power to disentangle the respective effects of HOMA-IR and FLI on conversion to NOD, or to better highlight the possible difference in AUC of their respective ROC curves for conversion to NOD (S2 Fig). In the same way, we also lack some statistical power to draw adequate mixed models in order to study the dynamics of the changes of FLI’s parameters during the follow-up regarding the risk of NOD. In addition, by design, we only focus here on patients with IFG and we don’t systematically perform OGTT to classify people with and without impaired glucose tolerance. Lastly, this exploratory study did not accurately take into account the inflation of the alpha risk, and the statistical significance of the results should be considered with caution.

Main strengths of our study were (1) the multi-centre approach and the duration of the follow-up, with a substantial number of events; (2) the quality and the frequency of the data collection, including a consultation with a physician and biological follow-up at least on a yearly basis, allowing timely detection of conversion to NOD; and (3) the robustness of our findings, FLI being positively associated with NOD, inversely associated with prediabetes reversion, and these results being steady in the different sensitivity analyses.

In summary, FLI is a practical, simple score that could help the physicians to better stratify the risk of conversion to NOD in patients with prediabetes. Its inverse association with prediabetes reversion, along with its expected covariation with FPG, makes it a potential candidate as a therapeutic education tool in the dialogue between the patient and the clinician. Further studies are needed to better characterize its relationship with insulin-resistance and its position as a biomarker in studies on prediabetes.

## Supporting information

S1 Fig Comparisons of ROC curves for NOD conversion using different prediction models. Full model (red curve) includes the diabetes risk score, FPG, A1C and FLI. The blue curves correspond to the same model after the exclusion of one of the components: diabetes risk score (A), FPG (B), A1C (C) and FLI (D). FLI: fatty liver index. FPG: Fasting Plasma Glucose.
(TIFF)

S2 Fig Baseline association between fatty liver index and HOMA-IR, before (A) and after (B) natural log transformation proposed using multivariate fractional approaches (n = 275).
(TIFF)

S3 Fig Receiver operating curves associated with the conversion to new onset diabetes during follow-up, for fatty liver index and HOMA-IR. Respective area under the curve: 63.7% (fatty liver index, grey curve) and 69.3% (HOMA-IR, black curve), p-value = 0.067. HMW: High Molecular Weight. HOMA-IR: Homeostasis model assessment of insulin resistance.
(TIFF)

S4 Fig Fatty liver index trajectory before the conversion to new onset diabetes or the end of follow-up. Black curve: population with conversion to new onset diabetes. Grey curve: population without conversion to new onset diabetes before the end of follow-up. For each year preceding follow-up, means are represented with 95% confidence intervals (point and vertical bar, respectively). The two curves are slightly shifted on the horizontal axis (± 0.05 year) for easy viewing purpose.
(TIFF)

S1 Table Characteristics of the whole IT-DIAB population and according to baseline fatty liver index values. (DOCX)

S2 Table Sensitivity analyses—Baseline characteristics of the population associated with conversion to new onset diabetes, using successively the HbA_1c_ criterion (N = 363), the event “confirmed NOD” (N = 358) and the ITDIAB population after exclusion of the 41 patients with “confirmed NOD” (N = 317). Survival approach—multivariate Cox analysis.
(DOCX)

S1 File STROBE statement–checklist of items that should be included in reports of *cohorts studies*. Applied here to the IT-DIAB study (NCT01218061).
(DOC)

S2 File IT-DIAB study protocol, English summary. (DOCX)

S3 File IT-DIAB study protocol, French version V8, 22 May 2013. (PDF)

## Abbreviations

ADA: American Diabetes Association
ECLIA: electrochemiluminescent enzyme immunoassay
FLI: Fatty Liver Index
FPG: Fasting plasma glucose
GGT: gamma glutamyl-transferase
HMW: High molecular weight
HOMA-IR: Homeostasis model assessment of insulin resistance
IDF: International Diabetes Federation
MetS: Metabolic syndrome
NAFLD: nonalcoholic fatty liver disease
NOD: new onset diabetes
OGTT: Oral glucose tolerance test
T2D: Type 2 diabetes
TG: Triglycerides
VLDL: very low density lipoprotein

## References

[1] Younossi Z, Anstee QM, Marietti M, Hardy T, Henry L, Eslam M, et al Global burden of NAFLD and NASH: trends, predictions, risk factors and prevention. Nat Rev Gastroenterol Hepatol. 2018;15:11–20. 10.1038/nrgastro.2017.109 28930295

[2] Yki-Järvinen H. Non-alcoholic fatty liver disease as a cause and a consequence of metabolic syndrome. Lancet Diabetes Endocrinol. 2014;2:901–910 10.1016/S2213-8587(14)70032-4 24731669

[3] Younossi ZM, Koenig AB, Abdelatif D, Fazel Y, Henry L, Wymer M. Global epidemiology of nonalcoholic fatty liver disease-Meta-analytic assessment of prevalence, incidence, and outcomes. Hepatology. 2016;64:73–84. 10.1002/hep.28431 26707365

[4] Donnelly KL, Smith CI, Schwarzenberg SJ, Jessurun J, Boldt MD, Parks EJ. Sources of fatty acids stored in liver and secreted via lipoproteins in patients with nonalcoholic fatty liver disease. J Clin Invest. 2005;115:1343–1351. 10.1172/JCI23621 15864352 PMC1087172

[5] Kotronen A, Westerbacka J, Bergholm R, Pietiläinen KH, Yki-Järvinen H. Liver fat in the metabolic syndrome. J Clin Endocrinol Metab. 2007;92:3490–3497. 10.1210/jc.2007-0482 17595248

[6] Ballestri S, Zona S, Targher G, Romagnoli D, Baldelli E, Nascimbeni F, et al Nonalcoholic fatty liver disease is associated with an almost twofold increased risk of incident type 2 diabetes and metabolic syndrome. Evidence from a systematic review and meta-analysis. J Gastroenterol Hepatol. 2016;31: 936–944. 10.1111/jgh.13264 26667191

[7] Park SK, Seo MH, Shin HC, Ryoo JH. Clinical availability of nonalcoholic fatty liver disease as an early predictor of type 2 diabetes mellitus in Korean men: 5-year prospective cohort study. Hepatology. 2013;57:1378–1383. 10.1002/hep.26183 23213066

[8] Anstee QM, Targher G, Day CP. Progression of NAFLD to diabetes mellitus, cardiovascular disease or cirrhosis. Nat Rev Gastroenterol Hepatol 2013;10:330–344 10.1038/nrgastro.2013.41 23507799

[9] Bedogni G, Bellentani S, Miglioli L, Masutti F, Passalacqua M, Castiglione A, et al The Fatty Liver Index: a simple and accurate predictor of hepatic steatosis in the general population. BMC Gastroenterol. 2006;6:33 10.1186/1471-230X-6-33 17081293 PMC1636651

[10] Yang BL, Wu WC, Fang KC, Wang YC, Huo TI, Huang YH, et al External validation of fatty liver index for identifying ultrasonographic fatty liver in a large-scale cross sectional study in Taiwan. PLoS ONE 2015;10(3): e0120443 10.1371/journal.pone.0120443 25781622 PMC4363626

[11] Koehler EM, Schouten JN, Hansen BE, Hofman A, Stricker BH, Janssen HL. External validation of the fatty liver index for identifying nonalcoholic fatty liver disease in a population-based study. Clin Gastroenterol Hepatol. 2013;11:1201–1204. 10.1016/j.cgh.2012.12.031 23353640

[12] Gastaldelli A, Kozakova M, Hojlund K, Flyvbjerg A, Favuzzi A, Mitrakou A, et al Fatty liver is associated with insulin resistance, risk of coronary heart disease, and early atherosclerosis in a large European population. Hepatology 2009;49:1537–1544. 10.1002/hep.22845 19291789

[13] Bae JC, Rhee EJ, Lee WY, Park SE, Park CY, Oh KW, et al Combined effect of nonalcoholic fatty liver disease and impaired fasting glucose on the development of type 2 diabetes: a 4-year retrospective longitudinal study. Diabetes Care. 2011;34:727–729. 10.2337/dc10-1991 21278140 PMC3041216

[14] Nishi T, Babazono A, Maeda T, Imatoh T, Une H. Evaluation of the fatty liver index as a predictor for the development of diabetes among insurance beneficiaries with prediabetes. J Diabetes Investig. 2015;6:309–316. 10.1111/jdi.12290 25969716 PMC4420563

[15] Franch-Nadal J, Caballeria L, Mata-Cases M, Mauricio D, Giraldez-Garcia C, Mancera J, et al Fatty liver index is a predictor of incident diabetes in patients with prediabetes: the PREDAPS study. PLoS ONE 2018;13(6):e0198327 10.1371/journal.pone.0198327 29856820 PMC5983533

[16] Lindström J, Tuomilehto J. The diabetes risk score: a practical tool to predict type 2 diabetes risk. Diabetes Care. 2003;26:725–731. 10.2337/diacare.26.3.725 12610029

[17] Matthews DR, Hosker JP, Rudenski AS, Naylor BA, Treacher DF, Turner RC. Homeostasis model assessment: insulin resistance and β-cell function from fasting plasma glucose and insulin concentrations in man. Diabetologia. 1985;28(7):412–419. 10.1007/bf00280883 3899825

[18] Alberti KGMM, Zimmet P, Shaw J, IDF Epidemiology Task Force Consensus Group. The metabolic syndrome—a new worldwide definition. The Lancet. 2005;366(9491):1059–1062. 10.1016/S0140-6736(05)67402-8 16182882

[19] Classification and Diagnosis of Diabetes: Standards of Medical Care in Diabetes. American Diabetes Association. Diabetes Care. 2018;41:S13–S27. 10.2337/dc18-S002 29222373

[20] Tilg H, Moschen AR, Roden M. NAFLD and diabetes mellitus. Nat Rev Gastroenterol Hepatol. 2017;14:32–42. 10.1038/nrgastro.2016.147 27729660

[21] Goessling W, Massaro JM, Vasan RS, D'Agostino RB Sr, Ellison RC, Fox CS. Aminotransferase levels and 20-year risk of metabolic syndrome, diabetes, and cardiovascular disease. Gastroenterology. 2008;135:1935–1944. 10.1053/j.gastro.2008.09.018 19010326 PMC3039001

[22] Lee DH, Ha MH, Kim JH, Christiani DC, Gross MD, Steffes M, et al Gamma-glutamyltransferase and diabetes—a 4 year follow-up study. Diabetologia. 2003;46:359–364. 10.1007/s00125-003-1036-5 12687334

[23] Fraser A, Harris R, Sattar N, Ebrahim S, Davey Smith G, Lawlor DA. Alanine aminotransferase, gamma-glutamyltransferase, and incident diabetes: the British Women's Heart and Health Study and meta-analysis. Diabetes Care. 2009;32:741–750. 10.2337/dc08-1870 19131466 PMC2660465

[24] Kim CH, Park JY, Lee KU, Kim JH, Kim HK. Fatty liver is an independent risk factor for the development of type 2 diabetes in Korean adults. Diabet Med 2008; 25:476–481. 10.1111/j.1464-5491.2008.02410.x 18346164

[25] Balkau B, Lange C, Vol S, Fumeron F, Bonnet F; Group Study D.E.S.I.R. Nine-year incident diabetes is predicted by fatty liver indices: the French D.E.S.I.R. study. BMC Gastroenterol. 2010;10:56 10.1186/1471-230X-10-56 20529259 PMC2898845

[26] Jäger S, Jacobs S, Kröger J, Stefan N, Fritsche A, Weikert C, et al Association between the Fatty Liver Index and Risk of Type 2 Diabetes in the EPIC-Potsdam Study. PLoS One. 2015;10:e0124749 10.1371/journal.pone.0124749 25902304 PMC4406732

[27] Prevention or Delay of Type 2 Diabetes: Standards of Medical Care in Diabetes—2019. Diabetes Care. 2019;42(Supplement 1):S29–S33. 30559229 10.2337/dc19-S003

[28] Sung KC, Wild SH, Byrne CD. Resolution of fatty liver and risk of incident diabetes. J Clin Endocrinol Metab. 2013;98:3637–3643. 10.1210/jc.2013-1519 23873989

