# Supplementary figures and images for: Fatty liver index is a strong predictor of changes in glycemic status in people with prediabetes: The IT-DIAB study
Source: PLoS One. 2019 Aug 29;14(8):e0221524. doi: 10.1371/journal.pone.0221524 (PMC6715190; doi:10.1371/journal.pone.0221524)

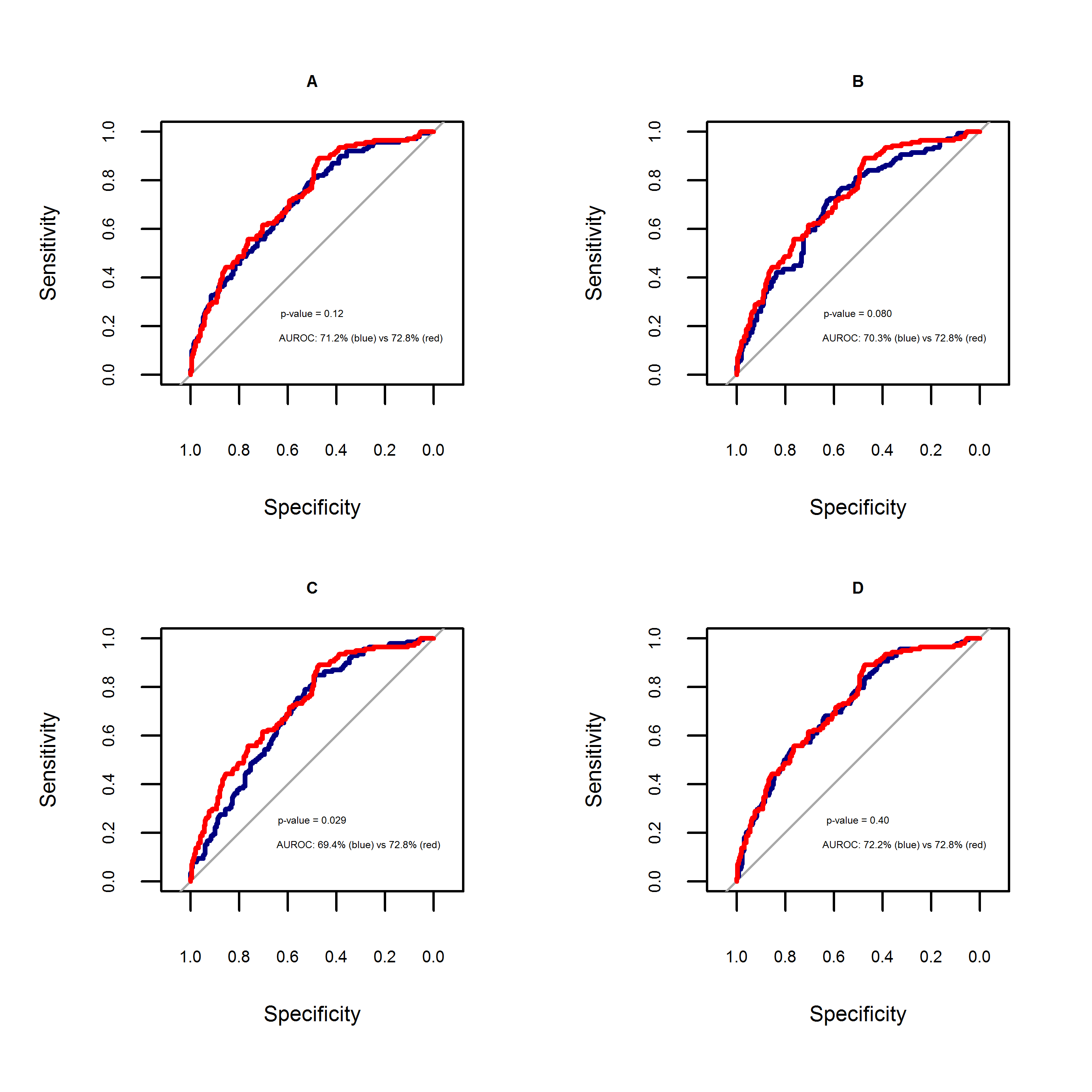

Supplement: S1 Fig — Full model (red curve) includes the diabetes risk score, FPG, A1C and FLI. The blue curves correspond to the same model after the exclusion of one of the components: diabetes risk score (A), FPG (B), A1C (C) and FLI (D). FLI: fatty liver index. FPG: Fasting Plasma Glucose. (TIFF) [file pone.0221524.s001.tiff]

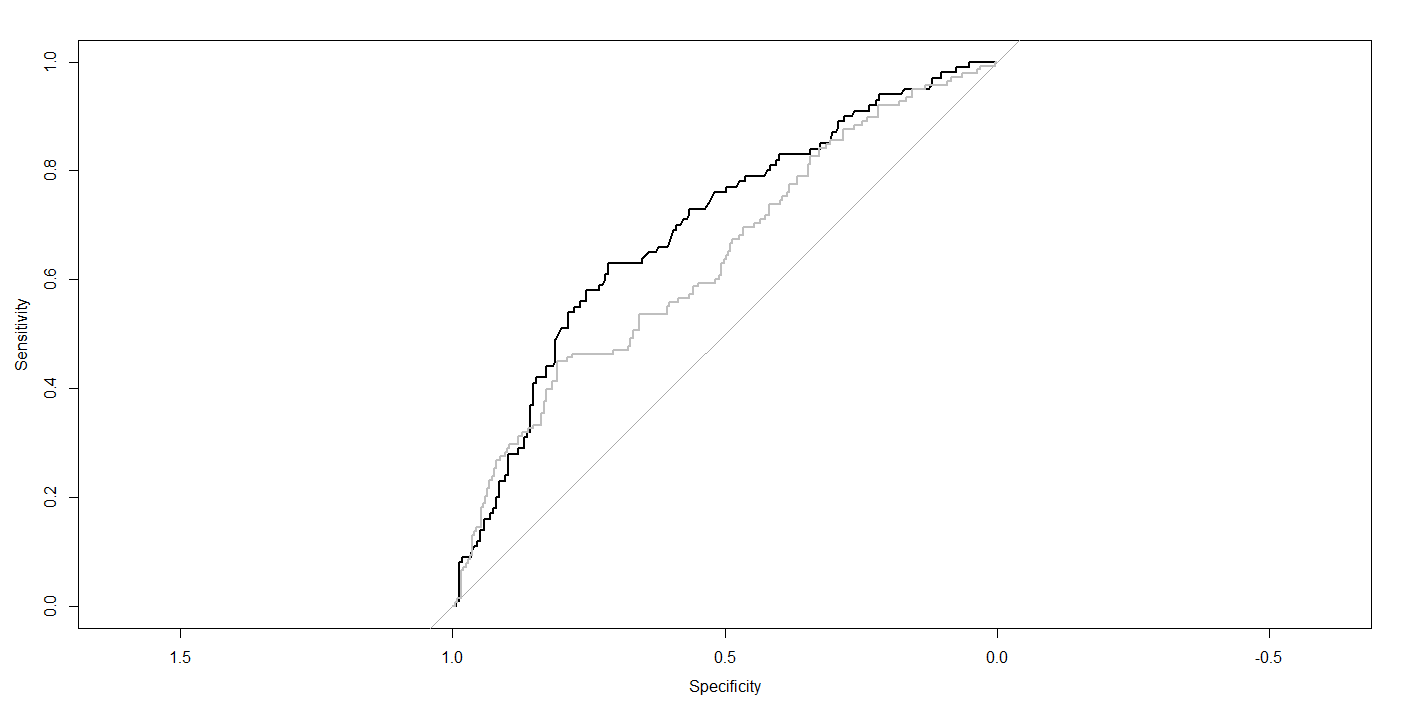

Supplement: S3 Fig — Respective area under the curve: 63.7% (fatty liver index, grey curve) and 69.3% (HOMA-IR, black curve), p-value = 0.067. HMW: High Molecular Weight. HOMA-IR: Homeostasis model assessment of insulin resistance. (TIFF) [file pone.0221524.s003.tiff]

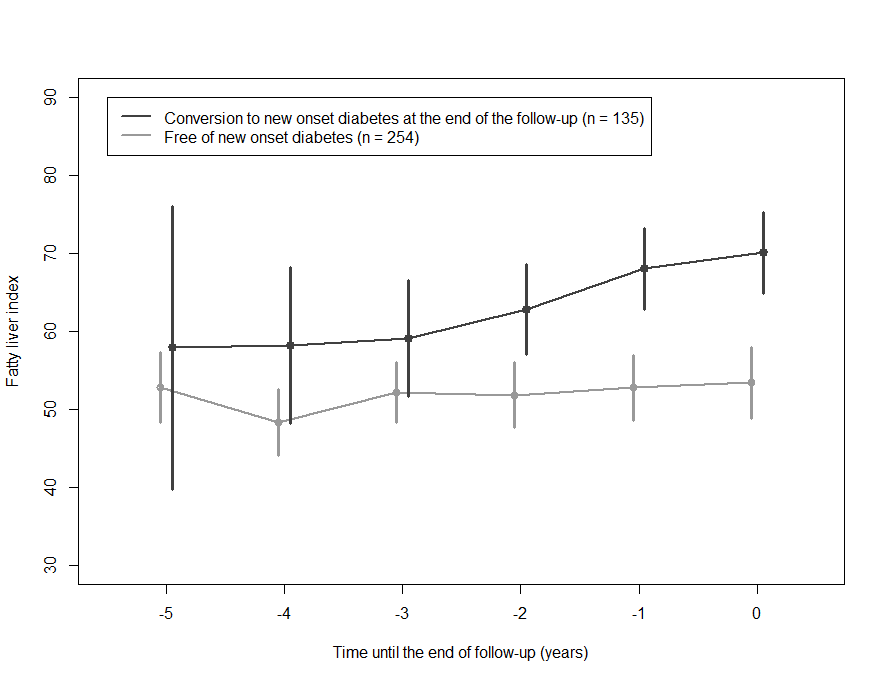

Supplement: S4 Fig — Black curve: population with conversion to new onset diabetes. Grey curve: population without conversion to new onset diabetes before the end of follow-up. For each year preceding follow-up, means are represented with 95% confidence intervals (point and vertical bar, respectively). The two curves are slightly shifted on the horizontal axis (± 0.05 year) for easy viewing purpose. (TIFF) [file pone.0221524.s004.tiff]
