# Supplementary material for: Fatty liver index is a strong predictor of changes in glycemic status in people with prediabetes: The IT-DIAB study
Source: PLoS One. 2019 Aug 29;14(8):e0221524. doi: 10.1371/journal.pone.0221524 (PMC6715190; doi:10.1371/journal.pone.0221524)
Supplement: S1 Table — (DOCX) [file pone.0221524.s005.docx]

**S1 Table. Characteristics of the whole IT-DIAB population and according to baseline fatty liver index values.**

|  | ***Baseline Fatty Liver Index*** | | | **Whole population**  **N = 389** |
| --- | --- | --- | --- | --- |
|  | **< 30**  **n = 77** | **30 ≤ FLI < 60**  **n = 103** | **≥ 60**  **n = 209** |  |
| *Clinical* |  |  |  |  |
| Sex (female) | 32 (41.6 %) | 33 (32 %) | 52 (24.9 %) | 117 (30.1 %) |
| Age (years) | 59.0 ± 10.4 | 59.4 ± 8.2 | 57.2 ± 10.2 | 58.1 ± 9.8 |
| Diabetes Risk Score | 10.8 ± 4.4 | 13.5 ± 4.3 | 15.8 ± 3.9 | 14.2 ± 4.5 |
| **Body mass index (kg/m²)** | 23.8 ± 2.5 | 27.4 ± 2.5 | 32.4 ± 5.5 | 29.4 ± 5.6 |
| **Waist circumference (cm)** | 83.1 ± 8.1 | 93.4 ± 5.6 | 107.4 ± 11.9 | 98.9 ± 13.9 |
| Hip circumference (cm) | 94.4 ± 6.3 | 99.8 ± 6.7 | 109.1 ± 13.1 | 103.7 ± 12.2 |
| Waist/Hip ratio | 0.88 ± 0.08 | 0.94 ± 0.06 | 0.99 ± 0.08 | 0.95 ± 0.09 |
| Hypertension | 20 (26 %) | 39 (37.9 %) | 99 (47.6 %) | 158 (40.7 %) |
| Metabolic syndrome | 13 (16.9 %) | 28 (27.2 %) | 155 (74.9 %) | 200 (51.7 %) |
| Statin therapy | 16 (20.8 %) | 23 (22.3 %) | 56 (26.8 %) | 95 (24.4 %) |
| *Biological (fasting)* |  |  |  |  |
| Plasma glucose (mg/dL) | 115 (113-119) | 115 (112-120) | 116 (113-120) | 115 (112-120) |
| HbA_1c_ (%) | 5.8 ± 0.4 | 5.8 ± 0.4 | 5.9 ± 0.4 | 5.8 ± 0.4 |
| HbA_1c_ (mmol/mol) | 39.9 ± 4.5 | 39.5 ± 4.3 | 40.7 ± 4.6 | 40.2 ± 4.5 |
| Fasting serum insulin (mUI/L) | 7.6 ± 5.0 | 10.5 ± 4.3 | 18.1 ± 10.0 | 14.1 ± 9.2 |
| HOMA-IR | 2.11 ± 1.48 | 2.99 ± 1.37 | 5.20 ± 2.97 | 4.02 ± 2.72 |
| Adiponectin (µg/mL) | 5.09 ± 3.15 | 3.92 ± 2.01 | 3.11 ± 1.57 | 3.70 ± 2.20 |
| AST (UI/L) | 20.5 ± 6.4 | 20.6 ± 5.0 | 25.2 ± 11.4 | 23.0 ± 9.4 |
| ALT (UI/L) | 22.1 ± 9.3 | 27.1 ± 11.7 | 37.1 ± 21.4 | 31.5 ± 18.4 |
| **GGT (UI/L)** | 22.7 ± 11.2 | 32 ± 18.2 | 60.3 ± 47.8 | 45.4 ± 40.1 |
| Total cholesterol (mg/dL) | 211.6 ± 31.7 | 209.7 ± 40.0 | 218.4 ± 40.1 | 214.8 ± 38.6 |
| **Triglycerides (mg/dL)** | 77.1 ± 29.4 | 109.4 ± 44.9 | 164.7 ± 85.1 | 132.7 ± 76.8 |
| LDL-c (mg/dL) | 131.3 ± 30.1 | 134.0 ± 35.9 | 137.2 ± 36.2 | 135.2 ± 35.0 |
| HDL-c (mg/dL) | 65.2 ± 15.8 | 53.6 ± 13.6 | 48.4 ± 12.2 | 53.1 ± 14.8 |
| Non-HDL-c (mg/dL) | 146.4 ± 32.3 | 156.1 ± 38.4 | 169.7 ± 40.6 | 161.5 ± 39.5 |
|  |  |  |  |  |
| *Outcomes* |  |  |  |  |
| Conversion to new onset diabetes | 15 (19.5 %) | 38 (36.9 %) | 85 (40.7 %) | 138 (35.5 %) |
| Confirmed conversion to new onset diabetes | 2 (2.8 %) | 9 (9.5 %) | 30 (15.6 %) | 41 (11.4 %) |
| Prediabetes reversion | 62 (80.5 %) | 71 (68.9 %) | 135 (64.6 %) | 268 (68.9 %) |

The four components of the *Fatty liver index* calculation are in bold.

**^†^** According to the International Diabetes Federation consensus statement.

FLI: fatty liver index. HOMA-IR: Homeostasis model assessment of insulin resistance; HMW: High Molecular Weight
