# Supplementary material for: Fatty liver index is a strong predictor of changes in glycemic status in people with prediabetes: The IT-DIAB study
Source: PLoS One. 2019 Aug 29;14(8):e0221524. doi: 10.1371/journal.pone.0221524 (PMC6715190; doi:10.1371/journal.pone.0221524)
Supplement: S2 Table — Survival approach—multivariate Cox analysis. (DOCX) [file pone.0221524.s006.docx]

**S2 Table. Sensitivity analyses - baseline characteristics of the population associated with conversion to new onset diabetes, using successively the HbA_1c_ criterion (N = 363), the event “confirmed NOD” (N = 358) and the ITDIAB population after exclusion of the 41 patients with “confirmed NOD” (N = 317). Survival approach - multivariate Cox analysis**

|  | **Multivariate Cox analysis** | | | | |
| --- | --- | --- | --- | --- | --- |
|  | **Before selection** | | | **After stepwise selection^†^** | |
|  | **HR [95% CI]** | ***p*-value** | | **HR [95% CI]** | ***p*-value** |
| **Study of conversion to NOD, using the HbA_1c_ criterion (127 events/363)** |  |  | |  |  |
| *Age (+1 SD)* | 0.92 [0.76; 1.11] | 0.38 | | - | - |
| *Sex (Women/men)* | 0.88 [0.58; 1.33] | 0.55 | | - | - |
| *Diabetes risk score (+1 SD)* | **1.43 [1.14; 1.80]**** | **0.002** | | **1.34 [1.09; 1.65]**** | **0.0053** |
| *Hypertension (y/n)* | 0.86 [0.59; 1.33] | 0.47 | | - | - |
| *Statin therapy (y/n)* | 0.96 [0.60; 1.43] | 0.84 | | - | - |
| *Fasting plasma glucose (+1 SD)* | **1.44 [1.20; 1.72]***** | **<0.0001** | | **1.43 [1.19; 1.71]***** | **0.00011** |
| *HbA_1c_ (+1 SD)* | **1.46 [1.15; 1.85]**** | **0.0018** | | **1.41 [1.14; 1.80]**** | **0.0021** |
| *Fatty Liver Index (+1 SD)* | 1.19 [0.95; 1.48] | 0.13 | | 1.23 [0.99; 1.51] | 0.058 |
|  | **Before selection** | | | **After stepwise selection^†^** | |
| **Study of confirmed conversion to NOD**  **(41 events/358)** | **HR [95% CI]** | | ***p*-value** | **HR [95% CI]** | ***p*-value** |
| *Age (+1 SD)* | 1.00 [0.70; 1.42] | | 0.98 | - | - |
| *Sex (Women/men)* | 0.99 [0.48; 2.05] | | 0.98 | - | - |
| *Diabetes risk score (+1 SD)* | **1.92 [1.26; 2.91]**** | | **0.0023** | **1.79 [1.23; 2.61]**** | **0.0024** |
| *Hypertension (y/n)* | 0.71 [0.35; 1.41] | | 0.33 | - | - |
| *Statin therapy (y/n)* | 1.08 [0.53; 2.21] | | 0.83 | - | - |
| *Fasting plasma glucose (+1 SD)* | **1.95 [1.41; 2.70]***** | | **<0.0001** | **1.95 [1.41; 2.69]***** | **<0.0001** |
| *HbA_1c_ (+1 SD)* | 1.04 [0.73; 1.49] | | 0.81 | - | - |
| *Fatty Liver Index (+1 SD)* | **1.63 [1.06; 2.50]*** | | **0.026** | **1.62 [1.08; 2.43]*** | **0.020** |
|  | **Before selection** | | | **After stepwise selection^†^** | |
| **Study of conversion to NOD after the exclusion of confirmed conversion to NOD**  **(77 events/317)** | **HR [95% CI]** | | ***p*-value** | **HR [95% CI]** | ***p*-value** |
| *Age (+1 SD)* | 0.87 [0.67; 1.11] | | 0.26 |  |  |
| *Sex (Women/men)* | 0.73 [0.42; 1.29] | | 0.28 |  |  |
| *Diabetes risk score (+1 SD)* | 1.23 [0.92; 1.65] | | 0.16 |  |  |
| *Hypertension (y/n)* | 1.13 [0.67; 1.92] | | 0.65 |  |  |
| *Statin therapy (y/n)* | 1.00 [0.56; 1.77] | | 0.99 |  |  |
| *Fasting plasma glucose (+1 SD)* | 1.25 [0.98; 1.59] | | 0.073 | 1.22 [0.96; 1.55] | 0.11 |
| *HbA_1c_ (+1 SD)* | **1.52 [1.16; 1.99]**** | | **0.0023** | **1.53 [1.19; 1.97]**** | **0.00093** |
| *Fatty Liver Index (+1 SD)* | 1.05 [0.80; 1.39] | | 0.71 | 1.20 [0.94; 1.52] | 0.14 |

HbA_1C_ criterion: same approach as for NOD in Table 3, but population with HbA_1c_ value ≥ 6.5% (47.5 mmol/mol) at baseline were excluded, and the criterion “first HbA_1c_ ≥ 6.5% (47.5 mmol/mol)” was added

**^†^**Considered candidates for multivariate adjusted Cox model were defined as follows: baseline fasting plasma glucose and Fatty liver index were “forced” in the model; age, sex, diabetes risk score, hypertension, statin therapy, HbA_1c_ were candidates. Multivariate Fractional Polynomial approach was also considered, but no polynomial transformation was proposed for a threshold selection value = 0.20

* *p-*value < 0.05; ** *p* < 0.01; *** *p* < 0.001
