# Supplementary material for: Fatty liver index is a strong predictor of changes in glycemic status in people with prediabetes: The IT-DIAB study
Source: PLoS One. 2019 Aug 29;14(8):e0221524. doi: 10.1371/journal.pone.0221524 (PMC6715190; doi:10.1371/journal.pone.0221524)
Supplement: S2 File — (DOCX) [file pone.0221524.s008.docx]

**IT-DIAB study protocol, English summary**

| Study title | IT-DIAB (Therapeutic innovation in Type 2 DIABetes) study |
| --- | --- |
| Keywords | Prediabetes / Type 2 diabetes (T2D) / beta-cell function / biomarkers |
| Research center | Nantes University Hospital |
| Study coordinator | Pr Bertrand CARIOU  Department of Endocrinology  L’institut du Thorax, Nantes University Hospital  Hôpital Guillaume et René Laënnec  Boulevard Jacques Monod  44093 Nantes Cedex 1, France  Tel : 00 33+(0) 2 53 48 27 07 / Fax : 00 33+(0) 2 53 48 27 08  Email : bertrand.cariou@univ-nantes.fr |
| List of the different centers | . Médecine du travail du CHU de Nantes  . Service de Médecine du travail de l’hôpital Saint-Jacques de Nantes  . Service d’Endocrinologie du CHU de Nantes  . Médecine du travail de DCNS Nantes  . Médecine du travail de la SEMITAN Nantes  . Médecine du Travail France Telecom Nantes  . Médecine du Travail de Nantes Métropole  . Médecine du Travail de la CAF de Nantes  . Etablissement Français du Sang Nantes  . Médecine du Travail de l’AMEBAT Nantes  . Médecine du Travail d’Arcelor Mittal  . Médecine du Travail d’Accenture Technology Solutions  . Médecine du Travail de La Poste  . Médecine du Travail de la Mairie de Nantes  . Centre d’Examens de Santé de Saint Nazaire  . Naturalpha, Nutrition Clinic Center, Lille |
| Study type | Primary care |
| Study agenda | **Total time:** 8 years  **Recruitment period:** 3 years  **Follow-up time for each patient:** 5 years |
| Study design | Multicentric  Observational  No randomization  Open-label  Prospective |
| Study objectives | **Primary objective:** To understand the pathophysiological mechanisms involved in the conversion to new onset diabetes (NOD) and to identify the new biomarkers for the risk of type 2 diabetes in a population of patients with prediabetes  **Secondary objectives:**   - To assess the prevalence of prediabetes in the Nantes area - To assess the place of HbA1C in the screening for prediabetes and T2D - To assess the interest of the Diabetes Risk score for the identification of people with a risk of conversion to NOD - To assess the prevalence of the other cardiovascular risk factors associated with prediabetes: dyslipidemia, high blood pressure and NAFLD - To constitute a biocollection |
| Expected population size | The objective is to recruit and follow-up 550 patients with prediabetes, identified by an impaired fasting glucose (IFG)  (fasting plasma glucose (FPG) ≥ 110 mg/dL and < 126 mg/dL)  or  (FPG ≥ 100 mg/dL and < 110 mg/dl and HbA_1c_ ≥ 6.5% (48 mmol/mol)) |
| Visits agenda | **Screening time**   - Screening for patients with high metabolic risk defined on a clinical score: Diabetes risk score ≥ 12 **or** history of IFG (FPG > 110 mg/dL and < 126 mg/dL)   **Inclusion**   - Blood sampling with FPG, HbA_1c_, lipid profile, creatinine and liver enzymes - If (FPG ≥ 110 mg/dL and < 126 mg/dL)   or  (FPG ≥ 100 mg/dL and < 110 mg/dl and HbA_1c_ ≥ 6.5% (48 mmol/mol))  => V_0_   - - Whole population: biocollection   - (Optional): 2H-Oral Glucose Tolerance Test (OGTT) in a subset of patients who consent   **Follow-up time**   - Planning for a 5-year annual follow-up (baseline (V_0_) - visit 5 (V_5_)) with   - Blood sampling: FPG, HbA_1c_, lipid profile, creatinine and liver enzymes + biocollection until V_5_   Thus, each patient will realize 6 visits (V_0_-V_5_) with 6 blood samples during 5 years |
| Main eligibility criteria, inclusion, non-inclusion and exclusion | Pre-inclusion criteria   - Diabetes Risk score ≥ 12 **or** history of impaired fasting glucose (FPG ≥ 110 mg/dL and < 126 mg/dL)   Inclusion criteria   - Men or women aged 18 or older - Affiliated to a social security scheme - Who have given their prior consent - With IFG at baseline visit: (FPG ≥ 110 mg/dL and < 126 mg/dL)   *or* ((FPG ≥ 100 mg/dL and < 110 mg/dl) and HbA_1c_ ≥ 6.5% (48 mmol/mol))  Non-inclusion criteria   - Type 2 diabetes mellitus or history of FPG ≥ 126 mg/dL - FPG < 100 mg/dL at baseline - FPG < 110 mg/dL and HbA_1C_ < 6.5% at baseline - History of treatment using antidiabetic drugs: metformin, glitazone, alpha-glucosidase inhibitors, sulfonylurea, repaglinide, DPP4-inhibitors or GLP-1 analogues - History of insulin therapy, except for gestational diabetes - Severe coagulation disorders - Thrombocytopenia < 100,000/mm^3^ - Severe renal insufficiency (defined using MDRD equation as eGFR<30 mL/min.1.73m^²^) - Severe liver impairment (prothrombin ratio <50%) - Severe psychiatric disorders - Alcohol abuse estimated >30 g/day - Patient’s opposition or inability to follow the study at least 5 years - Exclusion period of another clinical study - Minor or adult under guardianship - Deprivation of liberty by order of a competent court |
| Treatment, procedure, combination of study procedures | Except for the biocollection (blood samples), the acts are those which are realized annually for patients with prediabetes, according to French standards for primary care:   - Clinical examination (body weight, waist circumference, hip circumference) - Measurement of blood pressure - Blood sampling with the following biological test: FPG, HbA_1c_, lipid profile, creatinine, liver enzymes + biocollection until visit 5 |
| Main outcome | The main outcome will be the conversion to new onset diabetes (NOD), defined according to the WHO (World Health Organisation) as   - A FPG value ≥ 126 mg/dL controlled twice **or** - A 2H-PG after OGTT (75g) ≥ 200 mg/dL **or** - A random PG value ≥ 200 mg/dL associated with symptoms of T2D (weight loss, polyuria-polydipsia) |
| Secondary outcomes | The secondary outcomes will be:   - HbA_1C_ - Lipid profile: triglycerides, total cholesterol, HDL-c, LDL-c - Liver enzymes: AST, ALT, GGT, ALP - Creatinine - Blood pressure - Weight and body mass index - Waist circumference |
| Other analyses | Genetic, epigenetic, proteomic and blood dosing in order to identify new biomarkers of the risk of conversion to T2D in the IT-DIAB consortium |
| Statistical analyses | The main outcome being a better understanding of the pathophysiological mechanisms involved in the risk of conversion to new onset diabetes, we will use the common tools for statistical description (histogram, boxplot, median and interquartile range, variance/standard deviation, etc.) and multidimensional statistical exploration (principal component analysis, etc.). We will also be able to study the relationship between one and one ore more variables by means of correlation tests or multivariate regression analyses. |
