# Supplementary material for: Fatty liver index is a strong predictor of changes in glycemic status in people with prediabetes: The IT-DIAB study
Source: PLoS One. 2019 Aug 29;14(8):e0221524. doi: 10.1371/journal.pone.0221524 (PMC6715190; doi:10.1371/journal.pone.0221524)
Supplement: S3 File — (PDF) [file pone.0221524.s009.pdf]

Protocole IT-DIAB

N° d'enregistrement : 2010-A00240-39
Ref : PROG 09/11
Ref CPP : 2010-S2

« IT-DIAB (Innovation thérapeutique dans le diabète de type 2) : suivi prospectif d'une cohorte de patients pré-diabétiques sur 5 ans »

Investigateur Coordonnateur :
Pr Bertrand CARIOU
Clinique d'Endocrinologie, Maladies Métaboliques & Nutrition
L'institut du Thorax, CHU Nantes
Hôpital Guillaume et René Laennec,
Boulevard Jacques Monod
Saint-Herblain
44093 Nantes Cedex 1
tel : 02 53 48 27 07
fax : 02 53 48 27 08
email : bertrand.cariou@univ-nantes.fr

Méthodologiste :

Lucie Planche, Cellule de Promotion de la Recherche Clinique, CHU de Nantes

John Brozek, responsable ITDiab DataBank, Genfit

Etablissement responsable de la recherche :

CENTRE HOSPITALIER UNIVERSITAIRE DE NANTES

CHU de Nantes
Contact : Anne Omnès
Cellule de promotion à la recherche clinique
5, allée de l'île Gloriette
44 093 Nantes cedex 01 (FRANCE)
Contact :
Tel : 02 53 48 28 35
Fax : 02 53 48 28 36

Page de signature

SIGNATURE DU RESPONSABLE DE LA RECHERCHE

Le responsable de la recherche s'engage à réaliser cette étude en soins courants selon toutes les dispositions législatives et réglementaires dont pourrait relever la recherche et selon le protocole.		
**Nom et fonction du représentant signataire :**<br>Mme Anne OMNES<br>Responsable de la cellule de promotion de la recherche clinique<br>CHU de Nantes	**Date :**	**Signature :**

SIGNATURE DES INVESTIGATEURS

J'ai lu l'ensemble des pages du protocole de l'essai clinique dont le CHU de Nantes est le responsable de la recherche. Je confirme qu'il contient toutes les informations nécessaires à la conduite de l'essai. Je m'engage à réaliser l'essai en respectant le protocole et les termes et conditions qui y sont définis.<br>J'ai connaissance que la présente recherche s'inscrit dans le cadre des recherches en soins courants tels que définis par l'alinéa 2° de l'article L 1121-1 et l'article R 1121-3 du code de la santé publique. Les actes sont pratiqués et les produits sont utilisés de manière habituelle, mais des modalités particulières de surveillance sont prévues au travers de ce protocole.<br>Je m'engage à réaliser l'essai en respectant :<br>❖ les principes de la "Déclaration d'Helsinki",<br>❖ les règles et recommandations de bonnes pratiques cliniques internationales (ICH-E6) et française (règles de bonnes pratiques cliniques pour les recherches biomédicales portant sur des médicaments à usage humain - décisions du 24 novembre 2006)<br>❖ la législation nationale et la réglementation relative aux essais cliniques<br>❖ la conformité avec la Directive Essais Cliniques de l'UE [2001/20/CE]<br><br>Je m'engage également à ce que les investigateurs et les autres membres qualifiés de mon équipe aient accès aux copies de ce protocole et des documents relatifs à la conduite de l'essai pour leur permettre de travailler dans le respect des dispositions figurant dans ces documents.			
**Investigateur coordonnateur**	**Nom :**<br><br>**Pr Bertrand CARIOU**<br>Clinique d'Endocrinologie<br>Maladies Métaboliques & Nutrition,<br>Institut du Thorax,<br>CHU de Nantes	**Date :**	**Signature :**

LISTE DES ABREVIATIONS

AOMI	Artériopathie oblitérante des membres inférieurs
ARC	Attaché de Recherche Clinique
AVC	Accident vasculaire cérébral
BPC	Bonnes Pratiques Cliniques
CPP	Comité de Protection des Personnes
CNIL	Commission Nationale de l'Informatique et des Libertés
CCTIRS	Comité Consultatif sur le Traitement de l'Information en Matière de Recherche dans le Domaine de la Santé
CPRC	Cellule de Promotion de la Recherche Clinique
CRB	Centre de resources biologiques
CRF	*Case Report Form* (cahier d'observation)
DT2	Diabète de type 2
EAL	Exploration d'une anomalie lipidique
HbA1C	Hémoglobine Glyquée
HGPO	Hyperglycémie provoquée par voie orale
HTA	Hypertension artérielle
ICH	*International Conference on Harmonization* (Conférence internationale pour l'harmonisation)
IDE	Infirmière Diplômée d'Etat
IDF	International Diabetes Federation
IDM	Infarctus du myocarde
IFG	Impaired Fasting Glucose (hyperglycémie modérée à jeun)
IGT	Impaired glucose tolerance (intolérance au glucose)
INSERM	Institut National de la Santé et de la Recherche Médicale
TEC	Technicien d'Etude Clinique

Table des Matières

PAGE DE SIGNATURE 2
LISTE DES ABREVIATIONS 3
TABLE DES MATIERES 4
INTRODUCTION 6
1 JUSTIFICATION DE L'ETUDE 7
1.1 CONTEXTE EPIDEMIOLOGIQUE ET SOCIO-ECONOMIQUE DU DIABETE DE TYPE 2 7
1.2 HISTOIRE NATURELLE DU DT2 8
1.3 DEPISTAGE ET DIAGNOSTIC DES ETATS PRE-DIABETIQUES 10
1.4 PREVENTION CLINIQUE DU DIABETE DE TYPE 2 10
1.4.1 Les interventions hygiéno-diététiques 11
1.4.2 Les interventions pharmacologiques 11
1.4.3 Les limites actuelles des politiques de prévention et de dépistage du diabète de type 2 12
REFERENCES BIBLIOGRAPHIQUES 13
2 OBJECTIFS ET CRITERES DE JUGEMENT 15
2.1 OBJECTIF ET CRITERE D'EVALUATION PRINCIPAL 15
2.1.1 Objectif principal 15
2.1.2 Critère d'évaluation principal 15
2.2 OBJECTIFS ET CRITERES D'EVALUATION SECONDAIRES 15
2.2.1 Objectif(s) secondaire(s) 15
2.2.2 Critère(s) d'évaluation secondaire(s) 16
2.3 OBJECTIF ET CRITERES D'EVALUATION DES ETUDES ANCILLAIRES 16
3 DESIGN DE LA RECHERCHE 17
3.1 METHODOLOGIE GENERALE DE LA RECHERCHE 17
3.2 SCHEMA DE L'ETUDE. 17
4 POPULATION ETUDIEE 20
4.1 DESCRIPTION DE LA POPULATION 20
4.2 CRITERES DE PRE-INCLUSION 21
4.3 CRITERES D'INCLUSION 21
4.4 CRITERES D'EXCLUSION 21
5 DEROULEMENT DE L'ETUDE 23
5.1 TECHNIQUES D'ETUDES ET D'ANALYSES 23
5.1.1 Description détaillée des paramètres d'évaluation 23
5.1.2 Description des techniques et analyses 23
5.1.3 Liste des échantillons biologiques prélevés : 24
5.2 CALENDRIER DE L'ETUDE 25
5.3 IDENTIFICATION DE TOUTES LES DONNEES SOURCES NE FIGURANT PAS DANS LE DOSSIER MEDICAL 25
5.4 REGLES D'ARRET DE LA PARTICIPATION D'UNE PERSONNE 26
5.4.1 Critères d'arrêt prématuré de la participation d'une personne à la recherche 26
5.4.2 Procédures d'arrêt prématuré de la participation d'une personne à la recherche 26
5.4.3 Critères d'arrêt de la recherche (hors considérations biostatistiques) 26
6 DATA MANAGEMENT ET STATISTIQUES 27
6.1 RECUEIL ET TRAITEMENT DES DONNEES DE L'ETUDE 27
6.1.1 Recueil des données 27
6.1.2 Codage des données 27
6.1.3 Traitement des données 28
6.2 STATISTIQUES 28
6.2.1 Description des méthodes statistiques prévues, y compris du calendrier des analyses intermédiaires prévues 28
6.2.2 Justification statistique du nombre d'inclusions 29

6.2.3 Degré de signification statistique prévu ... 30
6.2.4 Critères statistiques d'arrêt de la recherche ... 30
6.2.5 Méthode de prise en compte des données manquantes, inutilisées ou non valides ... 30
6.2.6 Gestion des modifications apportées au plan d'analyse de la stratégie initiale ... 30
6.2.7 Choix des personnes à inclure dans les analyses ... 30
6.2.8 Randomisation ... 30

7 SECURITE ... 31

7.1 COMITE DE SURVEILLANCE INDEPENDANT ... 31
7.2 EFFET INDESIRABLE ... 31

8 ASPECTS ADMINISTRATIFS ET REGLEMENTAIRES ... 32

8.1 DROIT D'ACCES AUX DONNEES ET DOCUMENTS SOURCE ... 32
8.2 DONNEES INFORMATISEES ET SOUMISSION A LA CNIL ... 32
8.3 MONITORING DE L'ESSAI ... 32
8.4 CONSIDERATIONS ETHIQUES ... 32
8.4.1 Information du patient ... 32
8.4.2 Comité de Protection des Personnes ... 33
8.5 AMENDEMENTS AU PROTOCOLE ... 33
8.6 REGLES RELATIVES A LA PUBLICATION ... 33
8.7 DEVENIR DES ECHANTILLONS BIOLOGIQUES ... 33

LISTE DES ANNEXES ... 35

INTRODUCTION

L'incidence croissante du diabète de type 2 (DT2) dans nos sociétés pose un réel problème de santé publique à l'heure actuelle. Dans l'évolution naturelle de la maladie, le DT2 est précédée par une phase de pré-diabète, qui partage la même base physiopathologique, à savoir la combinaison d'une insulinorésistance et d'un défaut d'insulinosécrétion. L'objectif de ce projet est de mieux définir cet état pré-diabétique en suivant de façon prospective une cohorte de 500 sujets pré-diabétiques, et ce afin d'identifier de nouveaux marqueurs du risque de basculement du pré-diabète vers le DT2. A terme, une meilleure compréhension de l'évolution naturelle du pré-diabète devrait permettre de mieux cibler pour chaque sujet les stratégies thérapeutiques visant à prévenir l'apparition du DT2.

1 JUSTIFICATION DE L'ETUDE

1.1 Contexte épidémiologique et socio-économique du diabète de type 2

Touchant aujourd'hui près de 250 millions de personnes (i.e. 6% de la population mondiale), le diabète de type 2 progresse à une allure épidémique à travers le monde. Selon des prévisions de la Fédération Internationale du Diabète (IDF), sa prévalence devrait croître de 55% d'ici à 2025 pour affecter environ 380 millions de personnes (1). Le diabète touche tant les pays développés que les pays nouvellement industrialisés comme l'Inde ou la Chine ou les pays du Golfe. Les changements brutaux de régimes alimentaires associés au manque d'exercice physique des populations de plus en plus vieillissantes expliquent largement cette évolution. En parallèle, un phénomène inquiétant émerge dans les pays développés : le diabète de type 2 apparaît en effet à un âge de plus en plus précoce, y compris chez les moins de 15 ans où il progresse à un rythme de 3% par an. Cette « tendance lourde » est appuyée par de nombreuses études qui ont mis en évidence des liens étroits entre diabète et obésité : ainsi, environ 2/3 des diabétiques sont en surpoids ou obèses. Certains experts évoquent même le terme de « diabésité ». Le surpoids et l'obésité augmentent en effet, de façon spectaculaire dans les pays à faible ou moyen revenu, surtout en milieu urbain, atteignant des proportions épidémiques à travers le monde avec actuellement près d'1,6 milliards d'adultes en surpoids dont 400 millions cliniquement obèses. Surpoids et obésité représentent donc une des causes majeures de la progression du diabète de type 2 au niveau mondial.

Concernant la France, la publication récente du bulletin épidémiologique hebdomadaire fait état d'un taux de prévalence du diabète proche de 4% en 2008, qui dépasse les prévisions établies (2). On estime ainsi au minimum à 2 500 000 le nombre de diabétiques en France, dont vraisemblablement 500 000 à 1 000 000 qui s'ignorent. A noter l'existence de grandes disparités régionales avec des taux proches de 5% dans la région Nord-Pas-de-Calais, contre 3.2% en Pays de Loire (Figure 1).

L'amélioration de la prévention, du dépistage et du traitement du DT2 passe par une meilleure compréhension de sa physiopathologie, qui demeure complexe.

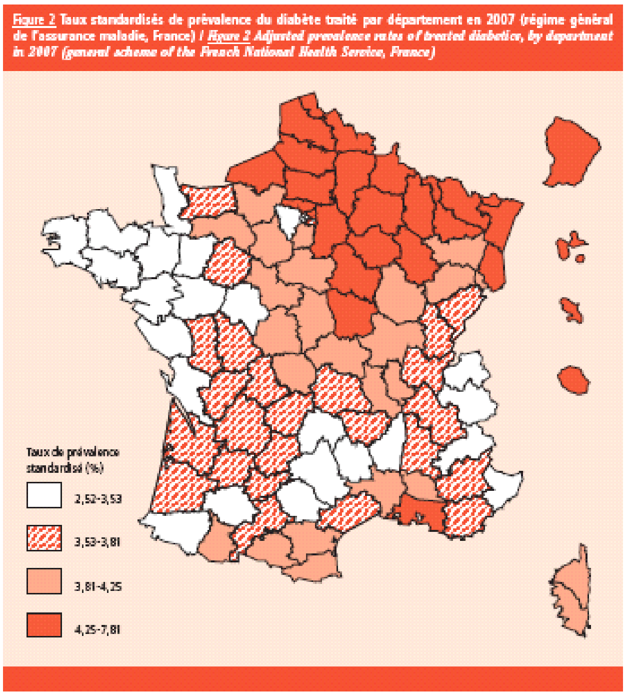

Figure 1 : Prévalence du diabète en France

(*Tiré de Kusnike-Joinville O. et al. BEH 12 nov 2008, N°43)*

1.2 Histoire naturelle du DT2

L'insuline est la seule hormone hypoglycémiante de l'organisme. Elle est produite par les cellules du pancréas. Elle favorise le stockage du glucose dans les tissus périphériques (muscles squelettiques et tissu adipeux), et réduit la production endogène de glucose par le foie (néoglucogénèse) (3). Chez un sujet sain, le pic de glucose plasmatique résultant de l'absorption du glucose alimentaire provoque la libération d'insuline par les cellules du pancréas. La quantité d'insuline ainsi produite permet de ramener rapidement la glycémie à une valeur normale par ses actions sur le foie et les tissus périphériques.

Les sujets « pré-diabétiques » présentent initialement une ***hyperglycémie modérée à jeun*** et/ou une ***intolérance au glucose*** dues à l'instauration progressive d'une résistance du foie et des tissus périphériques à l'action de l'insuline (*i.e.* insulinorésistance), ainsi qu'un déficit fonctionnel de la cellule béta pancréatique. Pour faire face à cette perte de sensibilité, le pancréas surproduit l'insuline, ce qui permet de ramener la glycémie à jeun à des valeurs normales. Parallèlement au développement de l'insulinorésistance, se produit une perte progressive de la masse fonctionnelle des cellules béta du pancréas. A terme, la perte de

masse fonctionnelle de cellules est telle que le pancréas n'est plus capable de compenser la perte de sensibilité à l'insuline, conduisant ainsi à l'apparition du DT2 (Figure 2).

Il est donc clair, que si l'on considère l'histoire naturelle du DT2, l'hyperglycémie peut être considérée comme une manifestation retardée d'un long processus physiopathologique s'étalant sur plusieurs années, partant de l'instauration d'une résistance à l'action de l'insuline, passant par une perte progressive de la masse fonctionnelle de cellules insulino-sécrétrices et conduisant à terme à une décompensation pancréatique à la base de l'hyperglycémie et du diagnostic de diabète (4-6).

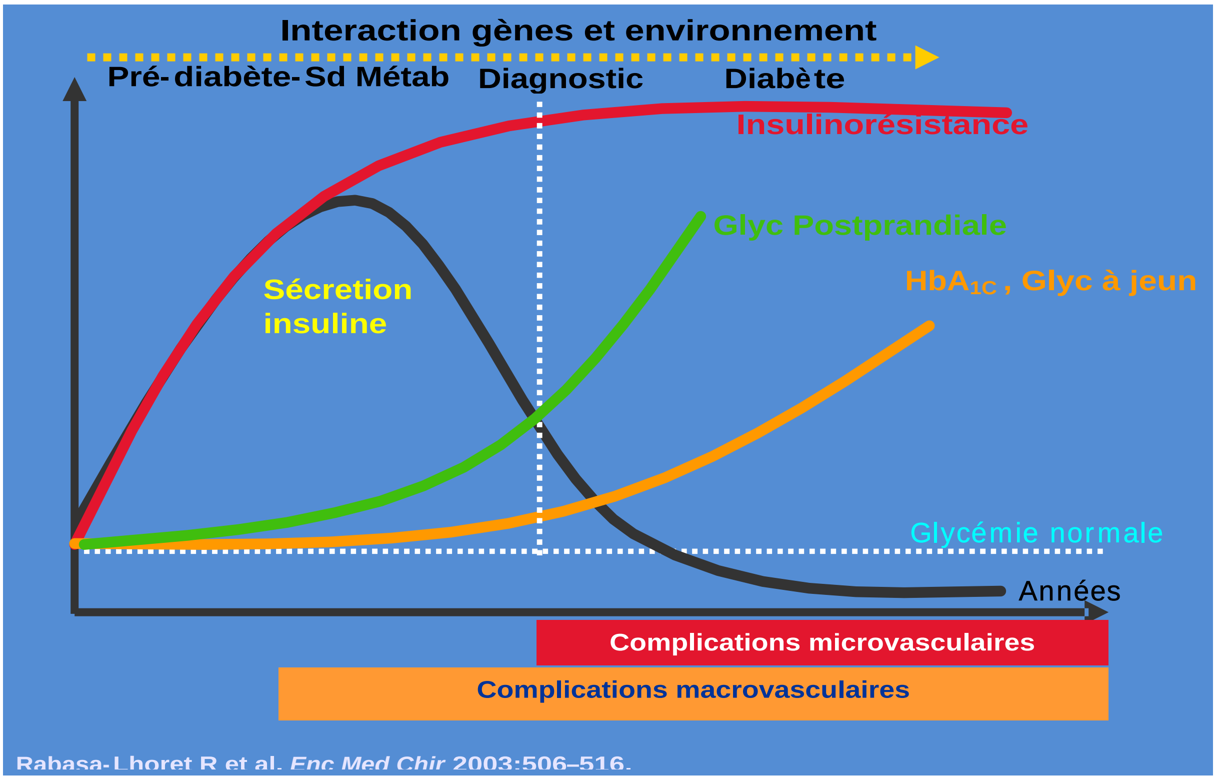

Figure 2 : Histoire naturelle du diabète de type 2

Il est important de noter que les complications cardiovasculaires (IDM, AVC, AOMI) qui font la gravité de la maladie surviennent dès le stade de pré-diabète, avant l'apparition de l'hyperglycémie franche (> 1.26 g/l) révélatrice du diabète. Dépister les sujets au stade de pré-diabète et prévenir le passage du pré-diabète au diabète de type 2 est donc un enjeu majeur de santé publique.

1.3 Dépistage et diagnostic des états pré-diabétiques

Le « pré-diabète » est un état intermédiaire entre une homéostasie du glucose normale et le DT2 avéré, incluant deux entités cliniques : l'hyperglycémie modérée à jeun (IFG : impaired fasting glucose) et l'intolérance au glucose (IGT : impaired glucose tolerance) (7).

L'hyperglycémie modérée à jeun est définie selon l'OMS par une glycémie à jeun ≥ 1.10 g/l, mais < 1.26 g/l.

L'intolérance au glucose est définie quant à elle par une glycémie 2H après une charge orale de 75 g de glucose ≥ 1.40 g/l et < 2 g/l.

Le risque de développer un DT2 est plus important chez les patients ayant une IFG et/ou une IGT, **le risque étant maximal chez ceux qui combinent les 2 anomalies**. Le risque de développer un diabète chez un sujet normoglycémique est de 0.7%/an. En revanche, ce risque est d'environ 5 à 10%/an chez les individus ayant une IFG et/ou une ITG. En l'absence d'intervention spécifique, la majorité de ces patients développent un diabète (8).

La place de l'hémoglobine glyquée (HbA1C) dans le dépistage et le diagnostic du diabète et des états pré-diabétiques reste discutée à l'heure actuelle. Il est important de noter que dans certains pays, comme notamment aux USA, l'HbA1C est validée pour le diagnostic du diabète (avec une valeur > 6.5% d'HbA1C) (17). En revanche, aucun consensus n'émerge actuellement concernant le pré-diabète avec des valeurs cibles d'HbA1C variant entre 5.7 et 6.4% dans les différentes études (18). De plus, il semble exister des différences ethniques pour le seuil d'HbA1C à retenir (19).

Afin de faciliter le dépistage des patients « pré-diabétiques », des scores cliniques de risque ont été développés. C'est le cas notamment du **Diabetes Risk Score** qui prend en compte des éléments de l'interrogatoire (antécédents familiaux de diabète, antécédents personnels d'HTA, activité physique, …) et des éléments cliniques (âge, poids, tour de taille) (cf Annexe 5). Ainsi, un score compris entre 13 et 20 (sur un total de 25) s'accompagne d'un risque de développer un DT2 de 32.7% à 10 ans (9, 10).

1.4 Prévention clinique du diabète de type 2

Au delà du dépistage, quelles sont les interventions thérapeutiques qui ont été évaluées pour prévenir ou ralentir l'apparition du DT2 chez des sujets pré-diabétiques ?

1.4.1 Les interventions hygiéno-diététiques

Dans la littérature, deux grandes études longitudinales ont évalué l'impact de mesures hygiéno-diététiques sur le basculement de la résistance à l'insuline vers le DT2 chez des patients obèses ou en surpoids :

– L'étude FDPS (Finnish Diabetes Prevention Study) portait sur 522 patients en surpoids ou obèses (BMI moyen de 31 kg/m2) en suivi longitudinal pendant 3,2 ans (11). Les sujets présentant une intolérance au glucose telle que définie par le WHO (World Health Organisation) étaient randomisés dans un groupe contrôle (quelques conseils portant sur le régime alimentaire et sur la nécessité d'exercices physiques quotidiens) ou dans un groupe faisant l'objet d'un suivi intensif (« coaching »): baisse de 30% de l'apport calorique journalier + exercice physique modéré (>150 minutes/semaine). Les résultats montrent que la proportion de patients évoluant vers le DT2 était significativement réduite dans le groupe faisant l'objet d'un contrôle strict par rapport au groupe contrôle (+3,2% par an vs + 7,8% par an). La perte de poids moyenne dans le groupe « intervention » était de 3,5 kg alors qu'elle n'était que de 0,8 kg dans le groupe contrôle. Au bout des 3,2 années (i.e. fin de l'étude), le risque d'évolution vers le DT2 était significativement réduit de 58% par rapport au groupe contrôle. Ce bénéfice se maintenait même après la fin de l'étude chez les patients ayant initialement bénéficié d'un suivi intensif (12).

- L'étude DPP (Diabetes Prevention Program) est l'étude longitudinale la plus large publiée à ce jour portant sur la prévention du DT2 chez des sujets présentant une intolérance au glucose à l'inclusion (13). Cette étude multiethnique confirme les résultats de FDPS. Les sujets présentant une intolérance au glucose étaient randomisés dans un groupe contrôle, dans un groupe d'intervention (réduction de l'apport calorique + exercice physique) ou dans un groupe traité par la metformine. Après une période de deux ans, une réduction de 58 % vs placebo du risque d'évolution vers le DT2 a été rapportée pour le groupe faisant l'objet d'un contrôle strict du mode de vie. Les résultats étaient comparables quelle que soit l'ethnie considérée. Dans tous les cas, cette réduction du risque d'évolution vers le DT2 s'accompagnait d'une réduction du poids corporel.

1.4.2 Les interventions pharmacologiques

1.4.2.1 La Metformine

L'essai clinique DPP mentionné précédemment évaluait les effets de la metformine sur la prévention du DT2 chez des patients présentant une intolérance au glucose (13). Le traitement par la metformine réduisait le risque d'évolution vers le DT2 de 31% par rapport au

groupe contrôle. Cependant, aucun bénéfice de la metformine n'a pu être mis en évidence chez les patients âgés (>60 ans) ou chez des patients en surpoids mais non obèses (BMI<30 kg/m2).

1.4.2.2 L'Acarbose

L'étude STOP-NIDDM évaluait les effets de l'acarbose (inhibiteur de l'alpha glucosidase qui diminue l'absorption intestinale du glucose) sur la progression vers le DT2 de patients présentant une intolérance au glucose (14). Les résultats montrent une réduction significative de 25% du risque d'évolution vers le DT2 chez les sujets traités quel que soit l'âge ou le BMI. Cependant les effets secondaires intestinaux de l'acarbose ont entraîné un arrêt de traitement chez 25% des sujets traités.

1.4.2.3 Les Thiazolidinediones

L'étude DREAM portant sur environ 5000 sujets évaluait les effets de la rosiglitazone sur l'évolution de patients intolérants au glucose. Une réduction très importante du risque d'évolution vers le DT2 a pu être mis en évidence (-60% par rapport au groupe contrôle)(15).

Récemment, l'étude ACTNOW a confirmé l'efficacité de la pioglitazone sur la prévention de l'apparition du DT2 dans une population de patients ayant une intolérance au glucose (16).

1.4.3 Les limites actuelles des politiques de prévention et de dépistage du diabète de type 2

A l'heure actuelle, la définition actuelle du « pré-diabète » ne répond pas aux critères de diagnostic indispensables à une prévention efficace du DT2 chez des patients à risque, car:

- La mesure en routine de l'intolérance au glucose n'est pas diffusable à large échelle en médecine générale pour des raisons pratiques et de coût.
- La mesure de l'intolérance au glucose n'est pas le reflet absolu du « pré-diabète » puisque 50-70 % des patients présentant une intolérance préalable au glucose ne développeront pas de DT2 dans les 5 ans qui suivent. Une extrapolation de l'étude DPP montre que environ 30% des sujets présentant une IGT à l'inclusion n'auront toujours pas développé de diabète 30 ans après.

Il apparaît donc indispensable de développer de nouveaux critères de diagnostic ou marqueurs du basculement du prédiabète vers le diabète, qui seraient plus performants dans l'identification des patients à très haut risque de basculement vers le DT2.

L'identification de ces nouveaux biomarqueurs se fera en collaboration avec plusieurs laboratoires de recherche académique, ainsi que des entreprises de biotechnologie spécialisées dans le domaine du diabète et des maladies métaboliques regroupées au sein d'un consortium de recherche (cf *Annexe 6*). L'intérêt et la force de ce projet sont que ces biomarqueurs seront validés de façon prospective dans une population cible de patients pré-diabétiques et qu'ils pourront être ainsi corrélés au risque de survenue du DT2.

Références bibliographiques

1. Zimmet P, Alberti KG, Shaw J 2001 Global and societal implications of the diabetes epidemic. Nature 414:782-787

2. Kusnik-Joinville O, Weill A, Salanave B, Ricordeau P, Allemand H 2008 Prevalence and treatment of diabetes in France: trends between 2000 and 2005. Diabetes Metab 34:266-272

3. Shulman GI 2000 Cellular mechanisms of insulin resistance. J Clin Invest 106:171-176

4. Matthews DR, Cull CA, Stratton IM, Holman RR, Turner RC 1998 UKPDS 26: Sulphonylurea failure in non-insulin-dependent diabetic patients over six years. UK Prospective Diabetes Study (UKPDS) Group. Diabet Med 15:297-303

5. Kahn SE 2001 The Importance of {beta}-Cell Failure in the Development and Progression of Type 2 Diabetes. J Clin Endocrinol Metab 86:4047-4058

6. Weyer C, Bogardus C, Mott DM, Pratley RE 1999 The natural history of insulin secretory dysfunction and insulin resistance in the pathogenesis of type 2 diabetes mellitus. J Clin Invest 104:787-794

7. Aroda VR, Ratner R 2008 Approach to the patient with prediabetes. J Clin Endocrinol Metab 93:3259-3265

8. Gerstein HC, Santaguida P, Raina P, Morrison KM, Balion C, Hunt D, Yazdi H, Booker L 2007 Annual incidence and relative risk of diabetes in people with various categories of dysglycemia: a systematic overview and meta-analysis of prospective studies. Diabetes Res Clin Pract 78:305-312

9. Lindstrom J, Tuomilehto J 2003 The diabetes risk score: a practical tool to predict type 2 diabetes risk. Diabetes Care 26:725-731

10. Silventoinen K, Pankow J, Lindstrom J, Jousilahti P, Hu G, Tuomilehto J 2005 The validity of the Finnish Diabetes Risk Score for the prediction of the incidence of coronary heart disease and stroke, and total mortality. Eur J Cardiovasc Prev Rehabil 12:451-458

11. Tuomilehto J, Lindstrom J, Eriksson JG, Valle TT, Hamalainen H, Ilanne-Parikka P, Keinanen-Kiukaanniemi S, Laakso M, Louheranta A, Rastas M, Salminen V, Uusitupa M 2001 Prevention of type 2 diabetes mellitus by changes in lifestyle among subjects with impaired glucose tolerance. N Engl J Med 344:1343-1350

12. Lindstrom J, Ilanne-Parikka P, Peltonen M, Aunola S, Eriksson JG, Hemio K, Hamalainen H, Harkonen P, Keinanen-Kiukaanniemi S, Laakso M, Louheranta A, Mannelin M, Paturi M, Sundvall J, Valle TT, Uusitupa M, Tuomilehto J 2006 Sustained reduction in the incidence of type 2 diabetes by lifestyle intervention: follow-up of the Finnish Diabetes Prevention Study. Lancet 368:1673-1679

13. Knowler WC, Barrett-Connor E, Fowler SE, Hamman RF, Lachin JM, Walker EA, Nathan DM 2002 Reduction in the incidence of type 2 diabetes with lifestyle intervention or metformin. N Engl J Med 346:393-403

14. Chiasson JL, Josse RG, Gomis R, Hanefeld M, Karasik A, Laakso M 2002 Acarbose for prevention of type 2 diabetes mellitus: the STOP-NIDDM randomised trial. Lancet 359:2072-2077

15. Gerstein HC, Yusuf S, Bosch J, Pogue J, Sheridan P, Dinccag N, Hanefeld M, Hoogwerf B, Laakso M, Mohan V, Shaw J, Zinman B, Holman RR 2006 Effect of rosiglitazone on the frequency of diabetes in patients with impaired glucose tolerance or impaired fasting glucose: a randomised controlled trial. Lancet 368:1096-1105

16. Defronzo RA, Banerji M, Bray GA, Buchanan TA, Clement S, Henry RR, Kitabchi AE, Mudaliar S, Musi N, Ratner R, Reaven PD, Schwenke D, Stentz FB, Tripathy D 2009 Actos Now for the prevention of diabetes (ACT NOW) study. BMC Endocr Disord 9:17

17. American Diabetes Association 2010 Diagnosis and classification of diabetes mellitus. Diabetes Care, 33 (supplt 1): S62-S69

18. Mostafa SA, Davies MJ, Srinivasan BT, Carey ME, Webb D, Khunti K 2010 Should glycated haemoglobin (HbA1c) be used to detect people with type 2 diabetes mellitus and impaired glucose regulation? Postgrad Med J 86: 656-662

19. Mostafa SA, Khunti K, Srinivasan BT, Webb D, Gray LJ, Davies MJ 2010 The potential impact and optimal cut-points of using glycated heamoglobin, HbA1c, to detect people with impaired glucose regulation in a UK multi-ethnic cohort. Diabetes Res Clin Pract 90: 100-108

2 OBJECTIFS ET CRITERES DE JUGEMENT

2.1 Objectif et critère d'évaluation principal

2.1.1 Objectif principal

L'objectif principal de cette étude est de suivre de façon prospective une cohorte de patients pré-diabétiques afin de comprendre les mécanismes physiopathologiques qui interviennent dans le basculement du pré-diabète vers le DT2 et d'identifier de nouveaux biomarqueurs du risque de DT2 dans cette population.

2.1.2 Critère d'évaluation principal

Le critère d'évaluation principale est la survenue d'un DT2 défini selon les critères diagnostiques de l'OMS :

- **une glycémie veineuse à jeun ≥ 1.26 g/l**
- **ou une glycémie veineuse aléatoire ≥ 2 g/l associée à des signes cliniques évocateurs d'un diabète** (amaigrissement, syndrome polyuro-polydypsique).
- une glycémie 2h après une charge orale en glucose (75g) ≥ 2.00 g/l, contrôlée à 2 reprises.

La glycémie à jeun sera réalisée à l'inclusion et annuellement pendant les 5 ans de suivi. A noter que l'HGPO ne sera pas réalisée systématiquement dans le suivi des patients, comme recommandé par l'HAS. Elle ne sera pratiquée que la première année (lors de la visite d'inclusion) chez les patients ayant une hyperglycémie modérée à jeun ou une HbA1C ≥ 6.5% associée à une glycémie à jeun comprise entre 1 et 1,1 g/L, et ce afin de dépister les patients ayant également une intolérance au glucose, voire un diabète de type 2.

2.2 Objectifs et critères d'évaluation secondaires

2.2.1 Objectif(s) secondaire(s)

Les objectifs secondaires de cette étude seront les suivants :

- Evaluer la prévalence du pré-diabète dans la région Nantaise

- Evaluer la place de l'HbA1C dans le dépistage du pré-diabète et du DT2
- Evaluer l'intérêt du *Diabetes Risk Score* (questionnaire d'évaluation clinique) dans l'identification des sujets pré-diabétiques.
- Evaluer la prévalence des autres facteurs de risque cardiovasculaires associés au pré-diabète : dyslipidémie, hypertension artérielle, hépatopathie métabolique.
- Evaluer l'impact des horaires et conditions de travail sur la prévalence des maladies métaboliques (pré-diabète, dyslipidémies…)
- Constituer au CHU de Nantes une collection d'échantillons biologiques destinée à la recherche sur le DT2

2.2.2 Critère(s) d'évaluation secondaire(s)

Les critères de jugements secondaires seront les paramètres suivants, ils seront évalués à l'inclusion, puis tous les ans pendant 5 ans :

- L'HbA1C
- Le profil lipidique (EAL): triglycérides, cholestérol total, HDL-cholestérol et LDL-cholestérol
- Les enzymes hépatiques : ALAT, ASAT, GGT, et PAL
- La créatinémie
- La pression artérielle
- Le poids et l'indice de masse corporelle (Poids (kg)/taille $(cm)^2$)
- Le tour de taille et le tour de hanche

2.3 Objectif et critères d'évaluation des études ancillaires

Non applicable

3 DESIGN DE LA RECHERCHE

3.1 *Méthodologie générale de la recherche*

La recherche présente les caractéristiques suivantes :
C'est une étude prospective de cohorte multicentrique cognitive.

3.2 *Schéma de l'étude.*

Le schéma de l'étude se déroule de la façon suivante (cf Figure 3) :

- **ETAPE 1** : Identification et recrutement des sujets à haut risque métabolique par les médecins investigateurs de l'étude sur les critères cliniques du *Diabetes Risk Score* (9) (cf *Annexe 5*).
- **ETAPE 2 :** Chez les sujets avec un *Diabetes Risk Score* ≥ 12 ou connus pour avoir une hyperglycémie modérée à jeun (i.e. glycémie à jeun ≥ 1.10 g/l et < 1.26 g/L), réalisation d'un dépistage du pré-diabète et des complications métaboliques par la réalisation d'une prise de sang avec le dosage de la glycémie à jeun, de l'HbA1C, du bilan lipidique, du bilan hépatique et de la créatinémie. En fonction du choix du sujet, cette prise de sang pourra être effectuée, soit dans un laboratoire de ville, soit dans le centre où il a été inclus quand une infirmière est sur place, soit dans le service d'Endocrinologie du CHU de Nantes.

  Il est à noter qu'un bilan sanguin datant de moins de deux mois à la date de sélection sera accepté pour l'inclusion dans l'étude, sous réserve qu'il comprenne la totalité des paramètres nécessaires à l'étude.
- **ETAPE 3 (optionnelle) :** Tous les sujets ayant soit une hyperglycémie modérée à jeun (≥ 1.10 et < 1.26 g/l) soit une HbA1C ≥ 6.5% associée à une glycémie à jeun ≥ 1 g/L et < 1,1 g/L se verront proposer une HGPO à 75g de glucose afin de dépister une intolérance au glucose. Comme précédemment, cette prise de sang pourra être effectuée soit dans un laboratoire de ville, soit dans le centre où il a été inclus quand une infirmière est sur place, soit dans le service d'Endocrinologie du CHU de Nantes.
- **ETAPE 4 :** les sujets ayant soit une hyperglycémie modérée à jeun soit une glycémie à jeun comprise entre 1 et 1,1 g/L associée à un taux d'HbA1C ≥ 6,5% auront une prise de sang en vue de la constitution de la sérothèque. Cette dernière sera effectuée par l'IDE-ARC en charge de ce projet soit dans le centre où le patient a été inclus soit dans le service d'Endocrinologie du CHU de Nantes.

- **ETAPE 5 :** Suivi annuel sur 5 ans pour les sujets ayant soit une hyperglycémie modérée à jeun soit une glycémie à jeun comprise entre 1 et 1,1 g/L associée à un taux d'HbA1C ≥ 6,5%. Chaque année les sujets inclus dans la cohorte auront une prise de sang pour la réalisation du bilan métabolique (idem étape 2) et de la sérothèque. Les sujets qui développeront un DT2 au cours de l'étude sortiront du suivi de l'étude. Si le diagnostic de diabète est porté en dehors des périodes programmées de suivi, le sujet bénéficiera, si possible, d'une dernière prise de sang pour la réalisation du bilan final. Leur prise en charge et leur suivi seront assurés par leur médecin généraliste, avec la possibilité d'un suivi conjoint par un endocrinologue du CHU de Nantes.

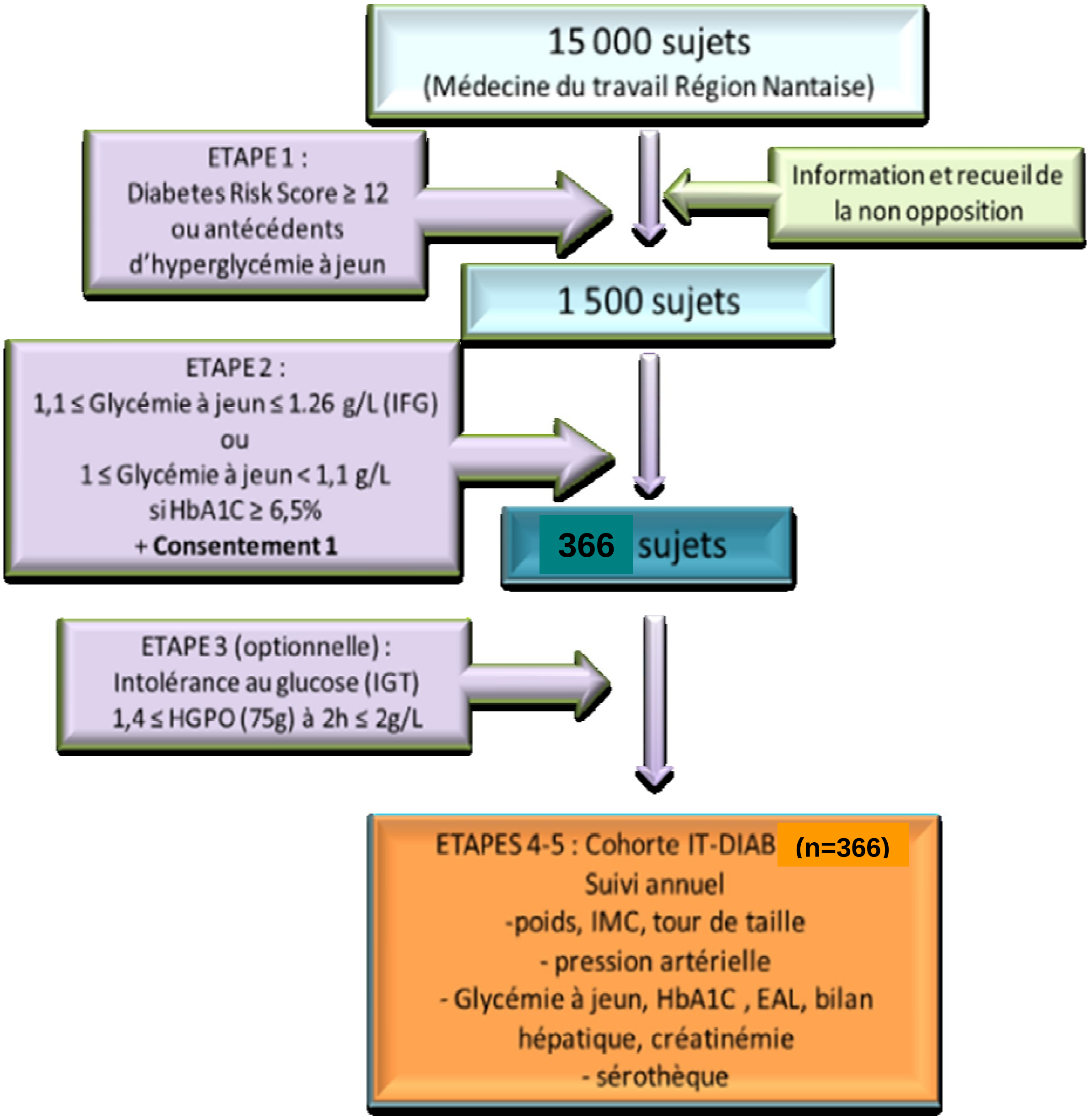

Figure 3 : Flow Chart de l'étude IT-DIAB

Il est important de préciser que les sujets « pré-diabétiques » identifiés dans cette étude bénéficieront tous d'une information diététique (équilibre alimentaire, réduction des apports en graisses, notamment saturées) et d'une sensibilisation à l'activité physique (Au minimum 2 X 30 min par semaine). A cette fin, des fiches d'information leurs seront remises par le médecin investigateur. En effet, ces règles hygiéno-diététiques ont fait la preuve de leur efficacité dans la prévention du DT2 chez les sujets à risque (11-13).

4 POPULATION ETUDIEE

4.1 Description de la population

La population étudiée concerne des sujets pré-diabétiques avec une hyperglycémie modérée à jeun ou une glycémie à jeun comprise entre 1 et 1,1 g/L si le taux d'HbA1C est ≥ 6,5%. L'originalité et l'objectif principal de ce travail résident dans le suivi prospectif des patients pendant 5 ans. A cette fin, nous avons choisi de recruter les sujets en majorité dans le cadre de la Médecine du travail car les patients peuvent bénéficier d'un suivi régulier.

Le recrutement sera fait dans plusieurs entreprises de la région nantaise, ce qui assure une base de recrutement de 15 000 personnes (cf annexe 3). En effet, nous anticipons devoir cribler environ 15 000 sujets pour identifier les 1500 avec un Diabetes Risk Score ≥ 12, ce qui nous fait au final un calcul d'effectif basé sur une prévalence du pré-diabète (i.e. glycémie à jeun ≥ 1.10 g/l) de 10% dans notre population. Ce chiffre représente, à nos yeux, une estimation pessimiste de la réalité, mais il devrait nous permettre d'atteindre notre objectif d'inclusions de 550 sujets. Sur ces 550 sujets pré-diabétiques, nous estimons qu'environ 100 développeront un DT2 dans les 5 ans du suivi.

Dans le cadre de la modification substantielle N°5, l'étude IT-DIAB présentant les mêmes objectifs et critères qu'une autre étude (Protocole DIABENORD N° ID-RCB : 2011-A00565-36 ; Ref CPP Amiens : 2011/24), il a été proposé de combiner les données des 2 études. L'objectif de 550 sujets est donc atteint en tenant compte des 366 patients au sein de l'étude IT-DIAB et des 207 patients au sein de l'autre étude.

L'information des sujets sera faite par le Médecin du travail ou Médecin investigateur. Une campagne de communication est également prévue dans la presse régionale pour sensibiliser les sujets au pré-diabète et au DT2. Des plaquettes d'information seront également réalisées et mises à disposition dans les salles d'attente des cabinets de la Médecine du travail et des autres centres associés.

Les sujets pourront, si ils le désirent, participer à une étude comprenant des investigations métaboliques plus poussées lors de journées d'explorations au CIC gastro-nutrition de Nantes : protocole DECODIAB (ref BRD 09/9-A, ref CPP : 2009-R27, n° Enregistrement : 2009-A00951-56). Les sujets participant aux études IT-DIAB et DECODIAB ne pourront pas participer simultanément à une autre recherche.

4.2 Critères de pré-inclusion

Le critère de pré-inclusion de cette étude est défini par le calcul du score de risque clinique : Diabetes Risk Score, qui identifiera les sujets à haut risque métabolique. Après le recueil de leur non opposition, les sujets ayant un score de risque ≥ 12 se verront proposer de participer à l'étude avec la réalisation du bilan sanguin de dépistage si aucun bilan sanguin complet (c'est à dire comprenant la totalité des paramètres nécessaires à l'étude) n'a été réalisé dans les 2 mois précédant la visite de sélection.

Les sujets ayant un score de risque < 12 mais connus pour avoir une hyperglycémie modérée à jeun (i.e. glycémie à jeun ≥ 1.10 g/l et < 1.26 g/L) pourront également être intégrés dans la cohorte après recueil de leur non opposition.

4.3 Critères d'inclusion

- Hommes ou femmes âgés de plus de 18 ans
- Affiliés au régime de sécurité sociale
- Sujets ayant donné leur consentement (non-opposition)
- Sujets ayant une hyperglycémie modérée à jeun : glycémie à jeun ≥ 1.10 g/l et < 1 .26 g/L

OU

- Sujets ayant une glycémie à jeun ≥ 1 g/l et < 1,1 g/L ET un taux d'HbA1C ≥ 6,5%.

4.4 Critères d'exclusion

- Diabète sucré ou glycémie à jeun ≥ 1.26 g/l
- Glycémie à jeun < 1.1 g/L ET taux d'HbA1C < 6,5%
- Glycémie à jeun < 1 g/L
- Sujets préalablement traités par des anti-diabétiques oraux : metformine, glitazones, inhibiteurs de l' α-glucosidase, sulfamides hypoglycémiants, repaglinide, inhibiteurs de la DPP-IV.
- Sujets préalablement traités par de l'insuline, à l'exception du diabète gestationnel
- Troubles sévères de la coagulation
- Thrombopénie < 100 000/mm^3
- Troubles psychiatriques sévères
- Insuffisance rénale sévère (Clairance de la créatinine < 30 ml/min)

- Insuffisance hépato-cellulaire sévère (TP < 50%)
- Consommation excessive d'alcool (> 30g/j)
- Sujet ne pouvant suivre l'étude pendant les 5 ans de suivi
- Sujet en période d'exclusion d'une étude précédente
- Sujet mineur ou majeur sous tutelle
- Sujet privé de sa liberté par décision judiciaire ou administrative

5 DEROULEMENT DE L'ETUDE

5.1 Techniques d'études et d'analyses

5.1.1 Description détaillée des paramètres d'évaluation

Les paramètres d'évaluation de cette étude sont des paramètres standards :

- **données anthropométriques** : poids (kg), taille (cm), tour de taille (cm), tour de hanche (cm), IMC (kg/m^2), pression artérielle (mm Hg), fréquence cardiaque (battements/min)
- **bilan biologique** :
  - glycémie à jeun (mmol/l)
  - HbA1C (%)
  - EAL : exploration d'une anomalie lipidique incluant le dosage des triglycérides plasmatiques (mmol/l), du cholestérol total (mmol/l), du HDL-cholestérol par mesure directe (mmol/l) et le calcul du LDL-cholestérol (mmol/l) par l'équation de Friedewald
  - bilan hépatique : aspartate amino transférase (ASAT), alanine amino transférase (ALAT), gamma glutamyltranspeptidase (GGT) et phospatases alcalines (PAL). Les valeurs seront exprimées en pourcentage par rapport à la valeur supérieure de chacun des dosages (% ukat).
  - créatinémie
  - Hyperglycémie provoquée par voie orale (HGPO) : glycémie (mmol/l) mesurée 2h après la prise orale de 75g de glucose.

5.1.2 Description des techniques et analyses

Le bilan d'inclusion (V0) sera réalisé soit dans un laboratoire de ville, soit dans le centre où le sujet a été inclus, soit dans le service d'Endocrinologie selon le choix du sujet et les possibilités du centre. L'HGPO et le prélèvement à V0 pour la constitution de la sérothèque seront faits dans un second temps selon les résultats du bilan biologique (*i.e.* uniquement chez les sujets ayant une hyperglycémie modérée à jeun ou une glycémie à jeun comprise entre 1 et 1,1 g/L ET un taux d'HbA1C $\geq$ 6,5%).

Les prélèvements sanguins des visites de suivi V1 à V5 (pour le bilan biologique et la constitution de la sérothèque) seront réalisés à jeun le matin par l'IDE-ARC en charge de l'étude dans les centres ou dans le service d'Endocrinologie du CHU de Nantes. Le prélèvement pour

la constitution de la sérothèque à V0 sera également réalisé à jeun le matin par l'IDE-ARC en charge de l'étude dans les centres ou dans le service d'Endocrinologie du CHU de Nantes.
Les prélèvements sanguins seront conservés à température ambiante (20 - 25°C), et acheminés dans les plus brefs délais (< 2h) au CRB de Nantes.

5.1.3 Liste des échantillons biologiques prélevés :

Pour chaque sujet à l'inclusion et lors de chaque bilan annuel, une série d'échantillons biologiques sera collectée :

- Pour le CRB (sérothèque en vue notamment d'analyses de protéomique + extraction ADN) :
  - 2 tubes héparine de 5ml
  - 2 tubes EDTA de 5ml
  - 2 tubes secs de 5 ml
  - 2 tubes citrate de 5ml
  - 1 tube EDTA de 5 ml pour l'extraction de l'ADN
- Pour la glycémie à jeun, la créatinémie, le bilan lipidique et le bilan hépatique: 1 tube sec de 8 ml
- Pour l'HbA1C : 1 tube EDTA de 4 ml

Le volume total de sang prélevé par journée d'exploration est donc de 57 ml.

La réalisation de l'hyperglycémie provoquée par voie orale (HGPO) consiste en l'administration orale d'une solution contenant 75g de glucose. Un prélévement sanguin (1 tube sec de 4 ml) est réalisé à jeun juste avant la prise de glucose (T0) et 2h après (T120min) pour le dosage de la glycémie. L'intérêt de l'HGPO est de pouvoir dépister les sujets intolérants au glucose qui sont plus à risque de développer un DT2 au cours du suivi (8).

5.2 Calendrier de l'étude

	Visite d'inclusion (V0)	Visite annuelle (V1, V2, V3, V4, V5)
Diabetes Risk score ≥ 12 ou antécédent d'hyperglycémie modérée à jeun (≥ 1.10 et < 1.26 g/l)	X	
Vérification critères inclusion/non-inclusion	X	
Information patients et recueil de la non opposition	X	
Courriel automatique d'inclusion	X	
Antécédents familiaux	X	
Antécédents personnels	X	
Examen clinique<br>Poids, taille, IMC<br>Tour de taille, tour de hanche<br>Pression artérielle	X	X
Analyses biochimiques :<br>Glycémie à jeun<br>HbA1C<br>Triglycérides, cholestérol total, HDL-cholestérol et LDL-cholestérol<br>ALAT, ASAT, GGT, PAL<br>Créatinémie	X	X
Hyperglycémie provoquée par voie orale (optionnelle)<br>Glycémie à T_0 & T_{120min}	X	
Sérothèque	X	X

5.3 Identification de toutes les données sources ne figurant pas dans le dossier médical

- Le résultat du Diabetes Risk Score
- Antécédents personnels et familiaux
- Traitements médicamenteux en cours
- Données morphométriques :

- Poids, taille & IMC
- Tour de taille, tour de hanches et rapport taille/hanches
- Fréquence cardiaque

• Pression artérielle (bras droit & gauche)

5.4 Règles d'arrêt de la participation d'une personne

5.4.1 Critères d'arrêt prématuré de la participation d'une personne à la recherche

• Décès du sujet
• Retrait du consentement
• Apparition d'un diabète de type 2 (critères OMS)
• Prescription d'antidiabétiques oraux
• Absence de suivi et de bilan complet (= examen clinique + glycémie à jeun + sérothèque)
• Sujet perdu de vue (ex : sujet ayant quitté l'entreprise et la région Nantaise).

5.4.2 Procédures d'arrêt prématuré de la participation d'une personne à la recherche

Pour la modalité et la durée du suivi des personnes ayant arrêté prématurément l'étude, se rapporter à la section statistique.

5.4.3 Critères d'arrêt de la recherche (hors considérations biostatistiques)

La durée de l'étude est estimée à 8 ans : 3 ans de recrutement des patients et 5 ans de suivi.
La fin prévue de l'étude aura lieu lors du dernier bilan du dernier sujet suivi.
Sur décision du CPP, du promoteur ou de l'investigateur coordonnateur, l'étude peut être interrompue (faute de recrutement, question devenue obsolète).
En cas d'arrêt prématuré de l'étude, l'information sera transmise dans un délai de 15 jours aux autorités compétentes (CPP).

6 DATA MANAGEMENT ET STATISTIQUES

6.1 Recueil et traitement des données de l’étude

6.1.1 Recueil des données

Un cahier d’observation électronique (eCRF) sera créé par sujet. Toutes les informations requises par le protocole doivent être fournies dans le CRF. Il reprendra les différentes étapes de la prise en charge du sujet dans le protocole. Il doit comprendre les données nécessaires pour confirmer le respect du protocole, déceler les écarts majeurs au protocole et toutes les données nécessaires aux analyses.

Les personnes responsables du remplissage des CRF (investigateurs, ARC…) devront être définies et seront identifiées dans le tableau des délégations des responsabilités de chaque centre (conservé dans le classeur investigateur).

Le recueil des données sera effectué directement par l’investigateur ou l’ARC en charge de l’étude, en utilisant un CRF électronique (eCRF) accessible depuis le site internet https://www.hugo-online.org/csonline et développé par la Cellule de Promotion du CHU de Nantes avec le logiciel Capture System de la société Clinsight l(version du pack Installé : 5.05.4102).

Chaque personne (investigateur, ARC/IRC, ARC de Promotion, ARC de Monitoring) disposera d’un compte utilisateur personnel en rapport avec son rôle (profil) qui lui aura été assigné par le promoteur de l’essai. La création et l’administration des comptes informatiques sera assurée par le Data Manager en charge de l’essai. Chaque utilisateur devra changer son mot de passe initial à la première connexion : mot de passe crypté de 8 caractères alphanumériques minimum, durée de validité du mot de passe : 30 jours, mise en veille après 15 minutes d’inactivité sur l’application, blocage du compte utilisateur après 3 échecs de connexion. Un guide de saisie et de navigation sera fourni à l’investigateur et à l’ARC/IRC chargés du recueil des données. Le Data Manager en charge de l’essai assurera une assistance téléphonique en cas de problème lié à l’utilisation de l’application Capture System ou du eCRF.

6.1.2 Codage des données

La première lettre du nom, la première lettre du prénom, et le numéro d’inclusion (code du centre investigateur + numéro du sujet) seront les seules informations qui figureront sur le cahier d’observation (CRF) et qui permettront de rattacher *a posteriori* le CRF au sujet.

En signant ce protocole l'investigateur principal et l'ensemble des co-investigateurs s'engagent à maintenir confidentielles l'identité des sujets qui ont participé à l'étude. Le responsable de la recherche est également tenu de coder les données des sujets sur tous les documents qu'il pourrait avoir en sa possession (compte-rendus d'examens d'imagerie, de biologie,) qui seraient joints au CRF.

6.1.3 Traitement des données

La collecte des données cliniques reposera sur la mise en place d'une base de données clinique et la création de masques de saisie à l'image du cahier d'observation en conformité avec le protocole et les réglementations actuellement en vigueur.
La structure de la base de données et des écrans de saisie sera approuvée par le responsable de la recherche de l'essai.

6.2 Statistiques

Responsable Statistique :
Lucie Planche, Cellule de Promotion de la Recherche Clinique, CHU de Nantes
John Brozek, responsable ITDiab DataBank, Genfit

6.2.1 Description des méthodes statistiques prévues, y compris du calendrier des analyses intermédiaires prévues

L'objectif principal étant de mieux comprendre les mécanismes physiopathologiques qui interviennent dans le basculement du pré-diabète vers le DT2, nous utiliserons pour décrire et étudier les données du métabolisme glucidiques l'ensemble des outils des statistiques descriptives (histogramme, boxplot, médiane, quartiles, variance, écart type, etc.) et des statistiques exploratoires multidimensionnelles (ACP, etc.). Nous pourrons également entreprendre l'étude de la liaison entre une et une à plusieurs variables par l'intermédiaire de tests de corrélation ou d'analyses par régression multivariées. Par exemple, nous pourrons entreprendre une analyse par régression logistique multivariée pour identifier les variables qui caractérisent le groupe de sujets "prédiabétiques qui basculent vers le DT2 » par rapport aux sujets "prédiabétiques qui ne basculent pas vers le DT2". Des analyses complémentaires pourront être réalisées à partir de variables secondaires tels que les taux sériques de triglycérides, de cholestérol, etc...

Dans un second temps, des analyses statistiques des données de protéomique vont s'attacher à répondre à deux questions : (i) la première concerne l'identification de différentes signatures protéiques entre nos groupes d'échantillons via des outils statistiques tels que le t-test, l'ANOVA, SAM, etc … (ii) nous pourrons mettre en œuvre des modèles particuliers afin d'appréhender l'association entre le profil protéique et certaines variables cliniques des patients telles que la glycémie à jeun pour identifier des marqueurs biologiques du DT2.

Toutes ces analyses permettront à terme, d'interpréter l'ensemble des informations biologiques, cliniques, et protéomiques pour confirmer ou générer de nouvelles hypothèses scientifiques de l'installation du pré-diabète et de son basculement vers le diabète de type 2.

6.2.2 Justification statistique du nombre d'inclusions

L'ensemble de la cohorte de sujets pré-diabétiques dans le cadre du programme IT-DIAB comportera **500 sujets suivis sur une durée de 5 ans**. Nous anticipons un taux de perdu de vue sur l'ensemble de l'étude de 10%. Nous prévoyons donc de recruter **550 patients** pour avoir au final une cohorte de 500 patients suivis sur 5 ans. Dans l'étude *DREAM*, 23% des patients criblés présentaient une intolérance au glucose et/ou une hyperglycémie modérée à jeun. Plus récemment, dans l'étude *ACT NOW*, 32% des sujets âgés de plus de 18 ans criblés avaient une intolérance au glucose. En revanche, il n'existe pas de données disponibles en France concernant la prévalence du pré-diabète. Il est probable que cette dernière soit inférieure à celle observée aux Etats-Unis, si l'on se fie à la plus forte prévalence de l'obésité et du DT2 dans ce pays. Afin d'augmenter la sensibilité du dépistage, nous ne réaliserons les glycémies à jeun que chez des sujets ayant un ***Diabetes Risk Score*** **≥ 12**, qui est associé à un risque de développer un diabète de type 2 compris entre 17 et 33% à 10 ans. Nous anticipons un taux de pré-diabète aux alentours de 35% dans la population chez les sujets ayant un score ≥ 12. Nous devrons donc réaliser des glycémies à jeun chez ≈ 1500 sujets pour identifier les 550 patients de la cohorte. Malgré l'absence de données épidémiologiques, nous anticipons devoir cribler environ 15 000 patients pour identifier les 1500 avec un Diabetes Risk Score ≥ 12, ce qui nous fait au final un calcul d'effectif basé sur une **prévalence du pré-diabète (i.e. glycémie à jeun ≥ 1.10 g/l) de 10%** dans notre population. Ce chiffre représente, à nos yeux, une estimation pessimiste de la réalité, mais il devrait nous permettre d'atteindre notre objectif d'inclusions de 550 patients.

Dans le cadre de la modification substantielle N°5, l'étude IT-DIAB présentant les mêmes objectifs et critères qu'une autre étude (Protocole DIABENORD N° ID-RCB : 2011-A00565-36 ; Ref CPP Amiens : 2011/24), il a été proposé de combiner les données des 2 études. L'objectif

de 550 sujets est donc atteint en tenant compte des 366 patients au sein de l'étude IT-DIAB et des 207 patients au sein de l'autre étude.

6.2.3 Degré de signification statistique prévu

Le degré de significativité prévu est de 5%.

6.2.4 Critères statistiques d'arrêt de la recherche

Non applicable

6.2.5 Méthode de prise en compte des données manquantes, inutilisées ou non valides

En cas de patient perdu de vue, les relances adéquates seront effectuées.
Nous anticipons un taux de perdu de vue sur l'ensemble de l'étude de 10%. Nous prévoyons donc de recruter 550 patients pour avoir au final une cohorte de 500 patients suivis sur 5 ans.

6.2.6 Gestion des modifications apportées au plan d'analyse de la stratégie initiale

Non applicable

6.2.7 Choix des personnes à inclure dans les analyses

Pour atteindre l'objectif principal de l'étude à savoir mieux comprendre les mécanismes physiopathologiques du basculement vers DT2, les populations analysées seront :

- Les patients qui ont développé un DT2
- Les patients dont la glycémie est restée stable ou a diminué tout au long du suivi

6.2.8 Randomisation

Non applicable

7 SECURITE

7.1 Comité de surveillance indépendant

Non applicable

7.2 Effet indésirable

La survenue d'un effet Indésirable lié à la prise en charge du patient au cours du présent protocole donnera lieu à une déclaration dans le système de vigilance adéquat (pharmacovigilance, biovigilance, hémovigilance, matériovigilance, etc…).

8 ASPECTS ADMINISTRATIFS ET REGLEMENTAIRES

8.1 Droit d'accès aux données et documents source

Les données médicales de chaque patient ne seront transmises qu'à l'organisme de rattachement de la personne responsable de la recherche ou toute personne dûment habilitée par celui-ci dans les conditions garantissant leur confidentialité.
Le cas échéant, l'organisme de rattachement de la personne responsable pourra demander un accès direct au dossier médical pour vérification des procédures et/ou des données de la recherche, sans violer la confidentialité et dans les limites autorisées par les lois et régulations.

8.2 Données informatisées et soumission à la CNIL

Les données recueillies au cours de l'étude seront conservées dans un fichier informatique respectant la loi « informatique et libertés » du 6 janvier 1978 modifiée en 2004. Cette recherche a fait l'objet d'une demande d'avis au CCTIRS, et d'autorisation à la CNIL (en cas d'étude monocentrique, seule la CNIL est consultée).

8.3 Monitoring de l'essai

Non applicable

8.4 Considérations éthiques

8.4.1 Information du patient

L'investigateur s'engage à informer le sujet de façon claire et juste du protocole (note d'information en annexe 2). Il remettra au sujet un exemplaire de la note d'information. Celle-ci précisera la possibilité pour le sujet de refuser de participer à la recherche.

8.4.2 Comité de Protection des Personnes

Le responsable de la recherche s'engage à soumettre le projet d'étude à l'autorisation préalable d'un Comité de Protection des Personnes (CPP). Les informations communiquées portent d'une part sur les modalités et la nature de la recherche et d'autre part, sur les garanties prévues pour les sujets participant à cet essai.

8.5 Amendements au protocole

Les demandes de modifications substantielles seront adressées par le responsable de la recherche pour avis au comité de protection des personnes concerné.

Le protocole modifié devra faire l'objet d'une version actualisée datée.
La note d'information devra faire l'objet de modification si nécessaire.

8.6 Règles relatives à la publication

Une copie de la publication sera remise au CHU de Nantes, responsable de la recherche de l'étude qui sera nécessairement cité. Les auteurs seront déterminés au prorata du nombre de patients inclus. L'investigateur coordonnateur établit la liste des auteurs.
Le protocole a été déclaré dans la base publique américaine : clinicaltrial.gov.

8.7 Devenir des échantillons biologiques

Afin de constituer une collection d'échantillons biologiques destinée à la recherche sur le pré-diabète et le diabète de type 2 (DT2), les prélèvements sanguins seront conservés à température ambiante (20 - 25°C), jusqu'à la centrifugation (à 1500g pendant 10min à 25°C). Les plasma/sérum seront transférés dans des cryotubes annotés par aliquots de 500µl et 200µl, puis ils seront rapidement congelés à -80°C. La préparation de ces échantillons sera réalisée à l'INSERM U915. Des fiches de traçabilité seront établies pour tous les échantillons.
Le CRB de Nantes assurera la conservation et la gestion (référencements, stockage, traçabilité des entrées, sorties ou incidents) des échantillons biologiques de la collection sous le contrôle d'un Comité de pilotage en vue de leur analyse ultérieure. Cette collection a fait l'objet d'une

déclaration, conformément à la réglementation en vigueur (D2008-402 au CPP Ouest II situé à Angers).

Les échantillons non utilisés dans le cadre de cette étude seront conservés après la fin de l'étude pendant une durée maximale de 10 ans par le CRB de Nantes. Ils permettront de réaliser, sous réserve d'une quantité suffisante d'échantillons, d'autres études sur le prédiabète et les facteurs de risque cardiovasculaires (dyslipidémies, HTA). Si le domaine de la recherche envisagée diffère du domaine initialement évoqué dans la note d'information du patient, il sera nécessaire d'obtenir un nouveau consentement du patient. L'utilisation d'échantillons de cette collection sera régie par un comité de pilotage de la collection (incluant un représentant du promoteur de l 'étude : le CHU de Nantes, l'investigateur principal : Pr Bertrand Cariou et le représentant du consortium IT-DIAB : société Genfit).

Liste des annexes

- Annexe 1 : Résumé de l’étude
- Annexe 2 : Note d’information patient
- Annexe 3 : Liste investigateurs
- Annexe 4 : Cahier d’observation
- Annexe 5 : Questionnaire Diabetes Risk Score
- Annexe 6 : Membres du consortium IT-DIAB
- Annexe 7 : Consentement de biocollection
